# CLIP‐170 spatially modulates receptor tyrosine kinase recycling to coordinate cell migration

**DOI:** 10.1111/tra.12629

**Published:** 2019-01-15

**Authors:** Kossay Zaoui, Stephanie Duhamel, Christine A. Parachoniak, Morag Park

**Affiliations:** ^1^ Department of Biochemistry McGill University Montreal Quebec Canada; ^2^ Rosalind and Morris Goodman Cancer Research Centre McGill University Montreal Quebec Canada; ^3^ Department of Medicine McGill University Montreal Quebec Canada; ^4^ Department of Oncology McGill University Montreal Quebec Canada

**Keywords:** cell migration, CLIP‐170, endocytosis, Met, trafficking

## Abstract

Endocytic sorting of activated receptor tyrosine kinases (RTKs), alternating between recycling and degradative processes, controls signal duration, location and surface complement of RTKs. The microtubule (MT) plus‐end tracking proteins (+TIPs) play essential roles in various cellular activities including translocation of intracellular cargo. However, mechanisms through which RTKs recycle back to the plasma membrane following internalization in response to ligand remain poorly understood. We report that net outward‐directed movement of endocytic vesicles containing the hepatocyte growth factor (HGF) Met RTK, requires recruitment of the +TIP, CLIP‐170, as well as the association of CLIP‐170 to MT plus‐ends. In response to HGF, entry of Met into Rab4‐positive endosomes results in Golgi‐localized γ‐ear‐containing Arf‐binding protein 3 (GGA3) and CLIP‐170 recruitment to an activated Met RTK complex. We conclude that CLIP‐170 co‐ordinates the recycling and the transport of Met‐positive endocytic vesicles to plus‐ends of MTs towards the cell cortex, including the plasma membrane and the lamellipodia, thereby promoting cell migration.

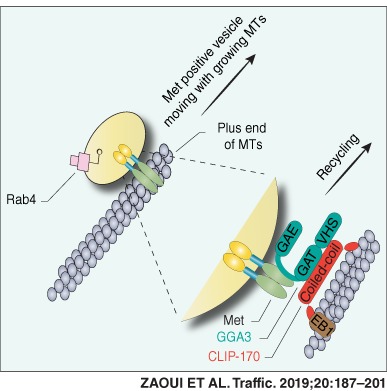

## 1 INTRODUCTION

Receptor tyrosine kinases (RTKs) control many aspects of cell behavior, including proliferation, survival, differentiation and migration in response to their environment. Upon ligand binding, RTKs become catalytically active and tyrosine phosphorylated, enabling the recruitment of proteins to initiate downstream signaling cascades. These processes are balanced by the simultaneous recruitment of endocytic proteins, which enhance RTK internalization, allowing for their removal from the cell surface and subsequent signal termination.^1^ Receptor internalization is now recognized as an important mechanism for regulating the signal transduction of functional receptors at the plasma membrane (PM).^1^, ^2^ Thus, a molecular understanding of the processes that modulate spatially restricted signaling of RTKs after internalization is key to our understanding of a biological response.

The Met RTK and its ligand, hepatocyte growth factor (HGF), play pivotal roles during development and adulthood by regulating epithelial to mesenchymal transitions of epithelial sheets and by promoting enhanced cell migration and invasion.^3^ Upon activation, the Met RTK is internalized and travels through endocytic compartments for degradation, or is recycled back to specific subcellular domains on the cell surface.^4^, ^5^ In addition to regulating signal termination, internalization and entry into endocytic compartments is an integral part of Met signaling, which controls strength, spatial and temporal restrictions of Met RTK signals.^6^, ^7^, ^8^, ^9^, ^10^, ^11^ Perturbation of Met trafficking on the degradative pathways leads to altered Met stability and signaling outputs implicated in the Met‐dependent tumorigenesis driven by increased Met abundance.^7^, ^12^, ^13^, ^14^

Recycling of RTKs to the cell surface can occur either directly from the early endosome via a “fast route” or indirectly through a “slow route,” traversing the endocytic recycling compartment.^15^ Fast recycling of the Met RTK is governed by recruitment of the monomeric adaptor protein, Golgi‐localizing, gamma‐adaptin ear homology domain, ARF‐binding protein 3 (GGA3).^8^ GGA3 modulates the entry of Met into a Rab4‐positive early recycling compartment where the recycling of Met is functionally required for cell migration in response to HGF.^8^ We recently identified new roles for GGA3 in regulating β1 integrin trafficking. We found that GGA3 knockdowns reduced total and cell surface levels of α2, α5 and β1 integrin subunits, inhibited cell spreading, and reduced focal adhesion numbers as well as cell migration.^16^

The microtubule (MT) cytoskeleton plays an essential role in numerous fundamental processes, including cell division, migration, differentiation, morphogenesis and intracellular trafficking.^14^, ^17^, ^18^, ^19^ MTs also serve as a rail on which motor proteins, such as kinesin and dynein convey their cargoes.^20^ Several interactions between MTs, dynein and membrane‐associated proteins have been associated with endosomal trafficking. Dynein is required for EGFR sorting and the morphogenesis of early endosomes^21^ and dynein LIC‐1 retrograde motor protein subunit has been reported to interact with Rab4A.^22^ MTs are also implicated in a rapid recycling pathways involving the transferrin receptor^23^ and play a role in the transport of epidermal growth factor (EGF) RTK‐positive endosomes through the plus‐end motor KIF16B,^21^, ^24^ implicating a role for MT‐based proteins in RTK transport. In mammalian cells, MTs are polarized with a slow‐growing minus‐end and a highly dynamic plus‐end alternating between phases of growth and shrinkage, towards the cell periphery.^25^, ^26^, ^27^ The functions of MTs are highly dependent on protein associations at their minus‐ and plus‐ends.^20^, ^28^ The MT plus‐end tracking proteins (+TIPs) are a structurally and functionally diverse group of proteins that accumulate at MT plus‐ends.^28^, ^29^ The cytoplasmic linker protein of 170 kDa (CLIP‐170) was the first +TIP identified,^30^ although more than 20 different +TIPs have now been characterized.^31^ These +TIPs contribute to loading cargo onto MTs for minus‐end‐directed transport towards the cell center.^32^, ^33^ CLIP‐170 has a key role in linking MTs to membrane trafficking.^34^ CLIP‐170 has been suggested to be critical for the migration of EGFR‐containing endosomes through a dynein‐driven transport.^35^ However, to date, our understanding of how the MT‐based machinery functions in endocytic trafficking of RTKs remains poorly understood.

Here, we provide evidence for the involvement of CLIP‐170 in the recycling of the Met RTK. We show that CLIP‐170 co‐ordinates the recycling and the transport of Met‐positive endocytic vesicles towards the plus‐end of MTs and at the cell cortex. We assign a functional role for CLIP‐170 in the recycling of the Met RTK through direct binding of CLIP‐170 to GGA3. We show that Met recycling is functionally required for the HGF‐dependent cell migration. These results link both endocytic and MT‐based processes in Met RTK‐mediated biological outcomes.

## 2 RESULTS

### 2.1 Met recycling requires the MT cytoskeleton and capture by the +TIP, CLIP‐170

**Figure 1:**
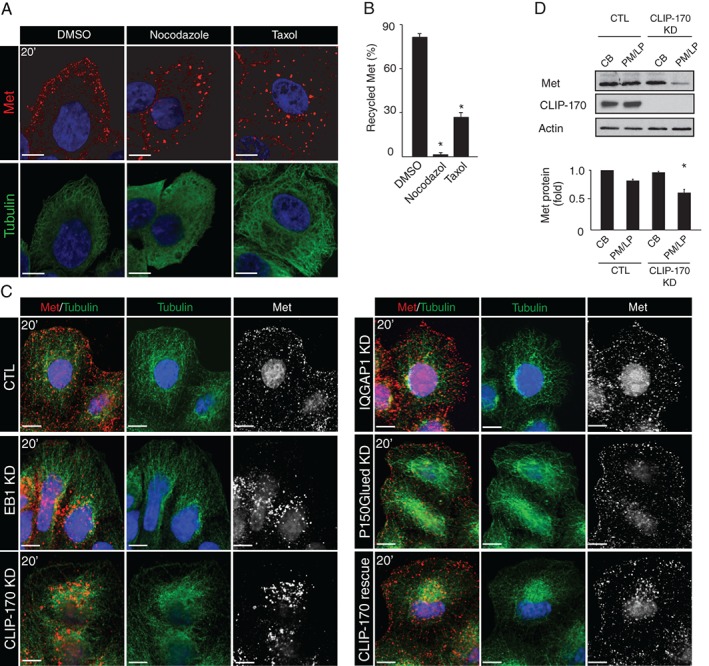
Met receptor trafficking to the cell cortex requires an intact MT cytoskeleton. (A) HeLa cells pre‐treated with DMSO or nocodazole (20 μM) for 10 minutes or taxol (0.1 μM) for 20 minutes were stimulated with 0.5 nM HGF (20 minutes). Top, cells were fixed and subjected to IF using anti‐Met and anti‐α‐tubulin antibodies. (B) Percentage of recycled Met from HeLa cells treated as in (A), was determined from IF images. (C) HeLa cells transfected with control (CTL), CLIP‐170, EB1, IQGAP1 or P150Glued siRNAs, were stimulated with HGF and processed for IF with anti‐α‐tubulin and anti‐Met antibodies. (D) Cell body (CB) and plasma membrane/lamellipodia (PM/LP) of HeLa cells transfected with CTL or CLIP‐170 siRNAs were fractionated. Equal amount of proteins was analyzed by western blot to reveal relative amounts of Met. Percentage of recycled Met from IF images was determined and quantified in the graph (bottom) (n = 3). Actin was used as loading control. Scale bar = 10 μm. *P < 0.05

Accumulating evidence supports a role for MTs in the recycling of cargo,^36^ including RTKs.^24^ To determine if MTs are required for Met localization to the cell cortex (PM and lamellipodia), cells were pre‐treated with the MT depolymerizing drug nocodazole or the stabilizing MT drug taxol. Treatment of cells with nocodazole or taxol abrogated HGF‐dependent Met recycling from endosomes back to the PM by 98.2% and 57.6%, respectively (Figures 1A,B and S1A,B). The +TIP, end‐binding protein 1 (EB1), is required for HGF‐induced cell extensions in three‐dimensional (3D) culture conditions and for vesicular trafficking to extensions.^37^ To identify if +TIP binding proteins are implicated in Met localization, we used an RNA interference‐based strategy to knockdown (KD) EB1. Depletion of EB1 decreased Met localization to the cell cortex in response to HGF (Figure 1C). EB1 depletion or dephosphorylation also results in CLIP‐170 delocalization from MTs (Figure S1C,D).^38^, ^39^ Hence the observed effects of EB1 KD on Met localization could be mediated through CLIP‐170. KD of CLIP‐170, but not other +TIP binding proteins, such as IQ motif containing GTPase activating protein 1 (IQGAP1) or P150Glued,^31^ decreased Met localization to the cell cortex in response to HGF (Figures 1C and S1C,D) and this was rescued by the re‐expression of a nontargetable CLIP‐170 expression construct (Figures 1C and S1E). To confirm this, we performed an immunofluorescence (IF)‐based recycling/localization assay.^8^ Following CLIP‐170 depletion, Met localization to the cell cortex in response to HGF was reduced from 80% to 10.7% (Figure S1F). A reduction in tyrosine phosphorylation of Met and its signal transducer, Gab1, were detected in CLIP‐170 KD cells after 20 minutes of HGF stimulation, consistent with a decreased recycling to the cell cortex (Figure S2A).^8^ Subcellular fractionations of PM/lamellipodia and cell body‐enriched fractions^40^ further confirmed that Met protein levels were decreased in the PM/lamellipodia fraction of CLIP‐170‐depleted cells (Figure 1D). Together, these data demonstrate that EB1, and its binding partner, CLIP‐170,^38^ are necessary for Met localization to the cell cortex.

The Rab family of small GTPases acts as molecular switches that spatially and temporally regulate vesicle transport.^41^ Met recycles to the cell cortex through a Rab4‐dependent pathway, which can be visualized using Alexa‐555‐labeled HGF.^8^ Twenty minutes post‐HGF stimulation, HGF/Met complexes entered into GFP‐Rab4‐positive recycling endosomes (Figure S2B; Movie S1). Growing ends of MTs are enriched in +TIP proteins^42^ and CLIP‐170. When overexpressed, CLIP‐170 accumulates predominantly to the plus‐ends of MTs.^30^ In fixed cells, Met and Rab4‐positive vesicles are enriched with CLIP‐170‐positive MT plus‐ends in response to HGF (Figure S2C).

**Figure 2:**
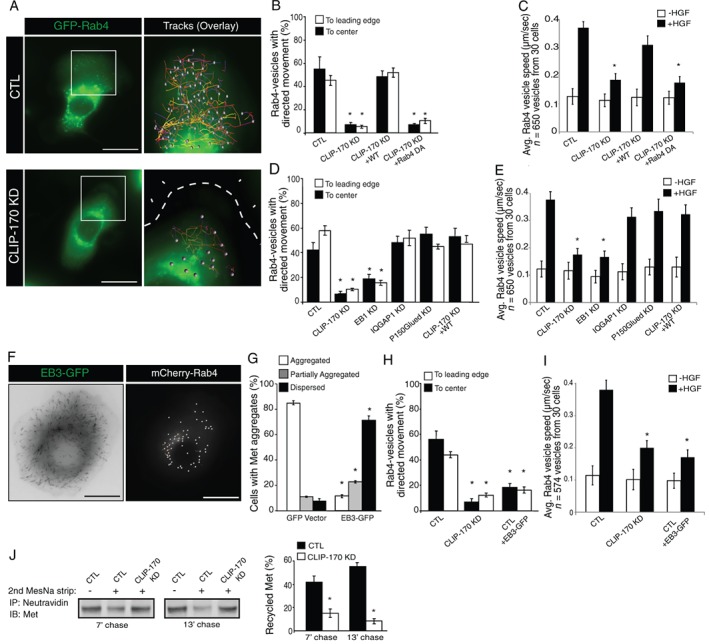
CLIP‐170 is required for HGF‐induced movement of Rab4‐positive vesicles and Met recycling. (A) SKBr3 cells co‐transfected with GFP‐Rab4 and CTL or CLIP‐170 siRNAs, were treated with 0.5 nM HGF (20 minutes). Insets show enlargement from the cell cortex. Arrowheads indicate the PM. Individual Rab4‐positive vesicles were tracked over time. The percentage of directed movement (B) and speed of vesicles (C) were analyzed. The percentage of directed movement (D) and speed of total vesicles (E) were quantified following depletion of CLIP‐170, EB1, IQGAP1 or P150Glued by siRNAs. (F) Overexpression of EB3‐GFP prevents binding of RFP‐CLIP‐170 to MT plus‐ends and alters HGF‐dependent movement of Rab4‐positive vesicles to cell periphery. (G) The data represent the percentage of cells with aggregated (white bars), partially aggregated (gray bars) or completely dispersed (black bars) Met‐positive vesicles at the MTs plus‐ends (see Figure S2J and Section 4 for details). The percentage of the directed movement (H) and the speed of vesicles (I) were quantified. (J) Left, CTL and CLIP‐170 KD cells were surface‐labeled on ice with Sulfo‐NHS‐SS‐biotin, stimulated 7 minutes with HGF at 37°C. Biotin from remaining cell surface receptors was removed by MesNa treatment at 4°C. Cells were then rewarmed to 37°C for the indicated times to allow recycling, followed by a second reduction with MesNa. Graph on the right shows the amount of recycled Met receptor expressed as the percentage of the pool of biotinylated Met during the internalization period, as described in Section 4. Scale bar = 10 μm. *P < 0.05

To understand the relevance of CLIP‐170 for Met trafficking, the localization and directionality of GFP‐Rab4‐positive vesicles in response to HGF was examined. In response to HGF, loss of CLIP‐170 resulted in the failure of most Rab4‐positive vesicles to reach the cell periphery and instead, localized to a more perinuclear compartment (Figure 2A). Additionally, the net directionality of vesicle movement was decreased (Figure 2B) and the average speed of Rab4‐positive vesicles was significantly reduced from 0.37 to 0.185 μm/s following CLIP‐170 KD (Figure 2C). Depletion of the +TIP, EB1, but not IQGAP1 or P150Glued, similarly impaired Rab4‐positive vesicle directionality in response to HGF (Figures 2D and S2D) and speed (Figure 2E), indicating a specific requirement of the two proteins in vesicular trafficking. Importantly, expression of a siRNA‐resistant CLIP‐170 construct restored Rab4 dynamics, confirming specificity of the KD (Figure 2B,C). In contrast, expression of a dominant‐active Rab4 construct was not sufficient for the rescue of HGF trafficking (Figure 2B,C). Notably, CLIP‐170 depletion did not reduce the overall mobility of all vesicles in response to HGF, as no detectable alterations in the dynamics of Rab11‐positive vesicles were observed (Figure S2E). Similar results were observed using single plan and spinning disc live cell imaging (Figure S2F‐I). Hence, CLIP‐170 has a specific effect on Rab4‐positive Met recycling vesicles.

As a complementary approach, we took advantage of EB3‐GFP construct overexpression that occludes and displaces the plus‐end binding of CLIP‐170 by inhibiting CAP‐GLY‐EEY/F interactions.^38^ Overexpression of EB3‐GFP alters Rab4‐positive vesicles to the cell cortex (Figure 2F). Overexpression of EB3‐GFP resulted in an approximate decrease of 50% in Met recycling (Figure S3A,B), abrogated Met‐positive vesicle aggregation at the MT plus‐ends (Figures 2G and S2J) and altered directionality (Figure 2H) and dynamics of Rab4‐positive vesicle movement in response to HGF (Figure 2I). These findings further support a requirement for specific CLIP‐170/MT‐interactions for efficient Met recycling to the cell cortex.

To confirm the role of CLIP‐170 on the Met recycling to the cell cortex (PM and lamellipodia), we tracked surface Met by a thiol‐cleavable amine‐reactive biotinylation reagent. Live HeLa cells were incubated with Sulfo‐NHS‐SS‐biotin at 4°C and internalization was started by incubating cells for 7 minutes with HGF at 37°C to allow accumulation of Met pools in early endosomes. During cell surface biotinylation and chase, CLIP‐170 KD cells exhibited a significant decrease in levels of Met returning to the cell surface to 15% compared to 42% in the control, following 7 minutes of HGF treatment; after 13 minutes of HGF treatment, levels of Met were decreased to 7% compared to 55% in the control (Figure 2J). This result revealed that CLIP‐170 plays an important role in Met recycling.

### 2.2 Recycling requires CLIP‐170 MT binding and formation of a Met/CLIP‐170 complex involving the CLIP‐170 coiled‐coil domain

**Figure 3:**
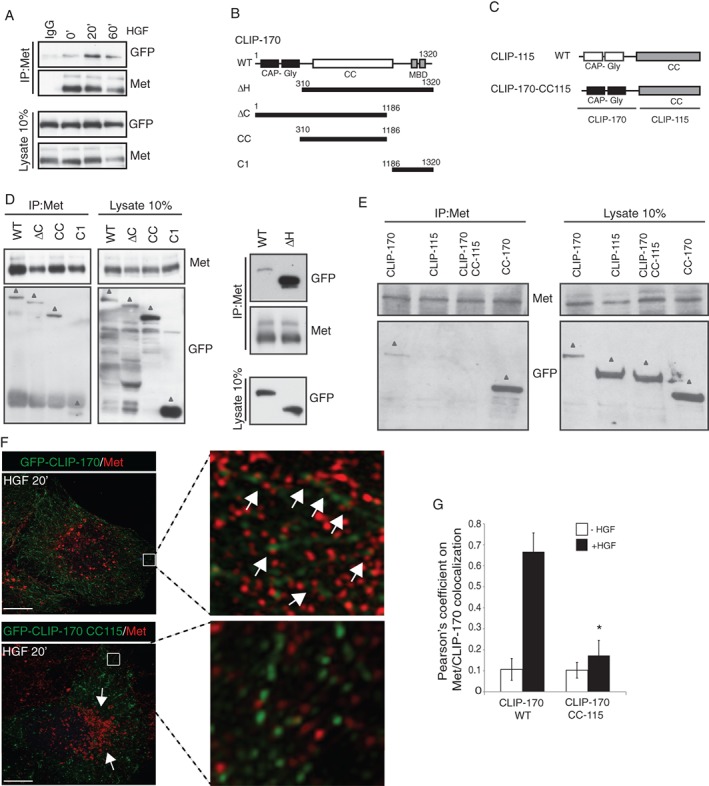
CLIP‐170 interacts with Met via its coiled‐coil domain. (A) HeLa cells were transfected with GFP‐CLIP‐170 and stimulated for the indicated times with HGF (0.5 nM). Protein lysates were subjected to IPs with Met or CTL IgG antibodies and coimmunoprecipitated proteins were separated by SDS‐PAGE and immunoblotted as shown. Schematic diagram of CLIP‐170 (B) and CLIP‐115 (C) mutant constructs. (D) HeLa cells transfected with GFP‐CLIP‐170 constructs shown in (B) were stimulated with HGF 0.5 nM, HGF (20 minutes) and then lysed. Proteins were subjected to IP with an anti‐Met antibody, and protein complexes were separated by SDS‐PAGE, transferred and immunoblotted as shown. (E) HeLa cells transfected with GFP‐CLIP‐170 and CLIP‐115 constructs shown in (C) were stimulated with HGF then lysed. Proteins were subjected to IP with an anti‐Met antibody. The bands of the proteins and mutants of interest are indicated by triangles. (F) 3D‐SIM image of HeLa cells transfected with GFP‐CLIP‐170 or the chimera GFP‐CLIP‐170 CC115 and stained with primary antibodies against GFP (green) or Met (red) and stained with DAPI (blue) as described in Section 4. (G) Graph on the right shows the Pearson's coefficient on Met/CLIP‐170 colocalization. Scale bar = 10 μm. *P < 0.05

CLIP‐170 is thought to act as a linker between endosomes and MTs contributing to secretory or endocytic trafficking.^32^, ^43^ To address the mechanism through which CLIP‐170 regulates recycling and localization of Met, we examined a potential physical association of the two proteins. Following overexpression, CLIP‐170 is strongly associated with the endogenous Met receptor in response to HGF stimulation (Figure 3A).

Because CLIP‐170 has not previously been implicated in interactions with RTK complexes, we utilized a structure‐function approach to define the domain(s) of CLIP‐170 required for association with Met. Removal of the N‐terminal MT‐binding CAP‐GLY (cytoskeleton‐associated protein‐glycine‐rich) motifs or head domain (ΔH), or deletion of the C‐terminal region (ΔC) of CLIP‐170 had little effect on the association of CLIP‐170 with Met (Figure 3B,D). Expression of the C‐terminus of CLIP‐170 alone (C1) was insufficient for association, whereas the central coiled‐coil (CC) domain of CLIP‐170 coimmunoprecipitated with Met (Figure 3B,D). The closest homolog of CLIP‐170 is CAP‐GLY domain containing linker protein 2 (CLIP‐115), which also contains a CAP‐GLY domain, a CC region and a short C‐terminal tail, and associates with MT plus‐ends and vesicles.^44^, ^45^ To examine the specificity, the ability of CLIP‐115 to coimmunoprecipitate with Met was examined. Under conditions where CLIP‐170 coimmunoprecipitates with Met, CLIP‐115 failed to coimmunoprecipitate (Figure 3C,E). Moreover, a fusion protein containing a substitution of the CC domain of CLIP‐115 (CC115) into CLIP‐170 failed to coimmunoprecipitate with Met (Figure 3C,E). Together, this data demonstrate that the Met receptor interacts specifically with CLIP‐170 via its CC domain.

To better characterize the Met/CLIP‐170 association and its role in Met recycling to the cell cortex, HeLa cells were transfected with GFP‐CLIP‐170 wild type (WT) or with the chimera GFP‐CLIP‐170 CC115 and stained with anti‐GFP and anti‐Met antibodies. We imaged and colocalized Met with CLIP‐170 WT or GFP‐CLIP‐170 CC115 after 20 minutes of HGF stimulation using the 3D structured illumination microscopy super‐resolution approach (see Section 4). We found that Met specifically accumulated with CLIP‐170 WT at the MTs plus‐end. However, the expression of GFP‐CLIP‐170 CC115 impaired Met distribution and induced a strong perinuclear accumulation of Met (Figure 3F).

**Figure 4:**
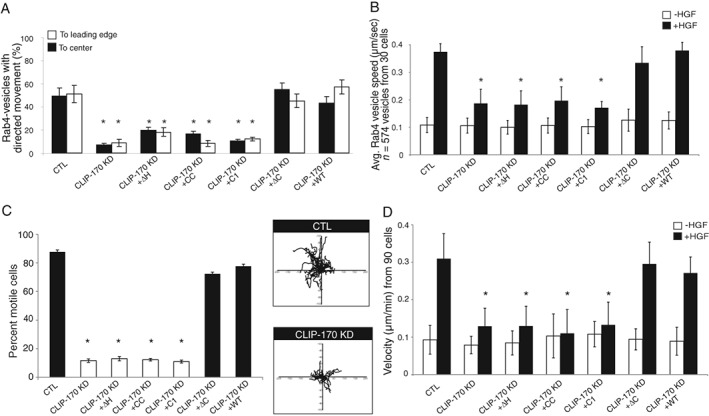
Interaction of CLIP‐170 with MTs and Met is required for Rab4 vesicle dynamics and HGF‐induced cell migration. Quantification of percentage of directed Rab4‐positive vesicles (A) and the speed vesicles (B) after expression of CLIP‐170 mutants in SKBr3 cells. (C) Left, percentage of moving HeLa cells from CTL or CLIP‐170 KD cells and rescue following expression of CLIP‐170 mutants. Right, analysis of cell migration paths in CTL and CLIP‐170 KD cells. Data represent the trajectories of 30 cells. (D) Cell velocity was determined by tracking cells (n = 3). *P < 0.05

The statistical analysis of Met colocalization with GFP‐CLIP‐170 CC115 revealed a significantly lower Pearson's coefficient, confirming the role of CLIP‐170 CC region in the interaction and Met recycling to the cell cortex (Figure 3G). Consistent with these coimmunoprecipitation studies, enhanced HGF‐dependent Rab4 vesicle dynamics and Met localization to the cell cortex, could be rescued by expression of siRNA‐resistant CLIP‐170 (WT) and (ΔC) constructs, but not by CLIP‐170 constructs lacking the ability to bind to MTs (ΔH) (Figures 4A,B and S3C). The CC domain of CLIP‐170, although sufficient for association with Met, failed to rescue HGF‐dependent enhanced mobility of Rab4‐positive vesicles and Met localization (Figures 4A,B and S3C). Together, these findings support a requirement for the MT binding head domain of CLIP‐170 for Met recycling, while the Met binding CC domain alone is required but not sufficient.

### 2.3 CLIP‐170 depletion abrogates HGF‐dependent cell motility and cell protrusion dynamics

HGF promotes lamellipodia formation and cell migration in epithelial cells through a process dependent on Met recycling.^8^ Consistent with a requirement for CLIP‐170 in Met recycling, only 10% of CLIP‐170‐depleted cells showed elevated migration in response to HGF (Figure 4C), where the net cell velocity was significantly reduced from 0.32 to 0.14 μm/min (Figure 4D). Importantly, re‐expression of CLIP‐170 (WT) or CLIP‐170 (ΔC), which rescues Met recycling to the cell cortex, restored HGF‐mediated cell migration. In contrast, re‐expression of the CLIP‐170 (ΔH) or (CC) mutants, which failed to bind MTs but could bind Met, failed to rescue HGF‐dependent cell migration (Figure 4C,D).

To address the importance of Met entry into Rab4‐positive vesicles for HGF‐induced cell motility, we abrogated Met recruitment to the PM through depletion of the Met‐specific recycling adaptor, GGA3.^8^ In response to HGF, GGA3 depletion resulted in 81% and 45% decrease in the outward mobility and speed of Rab4‐positive vesicles, respectively (Figure S3D‐F). These results were comparable to CLIP‐170 depletion (Figure S3D‐F). A corresponding decrease in overall cell motility from 0.267 μm/min in control cells to 0.145 μm/min in GGA3 KD cells was observed (Figure S3G). This data supports a requirement for entry of Met into the Rab4‐positive recycling pathway for CLIP‐170‐dependent localization of Met/Rab4‐positive vesicles to the cell periphery.

The protrusive activity of cell membranes was also altered in response to HGF in CLIP‐170 depleted cells (Figure S4A‐E). To understand how CLIP‐170 couples with regulators of the actin remodeling machinery at the cell cortex in response to HGF, we investigated by IF and live cell microscopy the subcellular localization of Arp3 and VASP, which are key regulators of filopodia and lamellipodia formation. While Arp3 and VASP are localized to the cell cortex in control cells, their localization is mainly limited to the cytoplasm in CLIP‐170 depleted cells (Figure S4A,B). These less stable membrane protrusions observed in CLIP‐170‐depleted cells corresponded to a quantitative reduction in overall protrusion size by 40% in lamellipodia width using actin‐mCherry, by 60% and 66.5% in the width and length of protrusion, respectively, and a decrease by 58% in the length of filopodia (Figure S4C,D). Kymograph analyses revealed that in response to HGF, control cells formed protrusive membrane edges that extended in a smooth, stable and persistent forward direction (Figure S4E). Edge phase density remained low, indicating that membranes did not show cycles of extension and retraction. In contrast, following depletion of CLIP‐170, protrusive membrane edges were less regular, with fluctuating edge phase density, indicating that membranes were dynamically extending and retracting (Figure S4E). Taken together, these results indicate that CLIP‐170 is essential for HGF‐dependent cell extension and migration.

### 2.4 CLIP‐170 regulates specifically Rab4 vesicle mobility and HGF‐dependent cell migration

**Figure 5:**
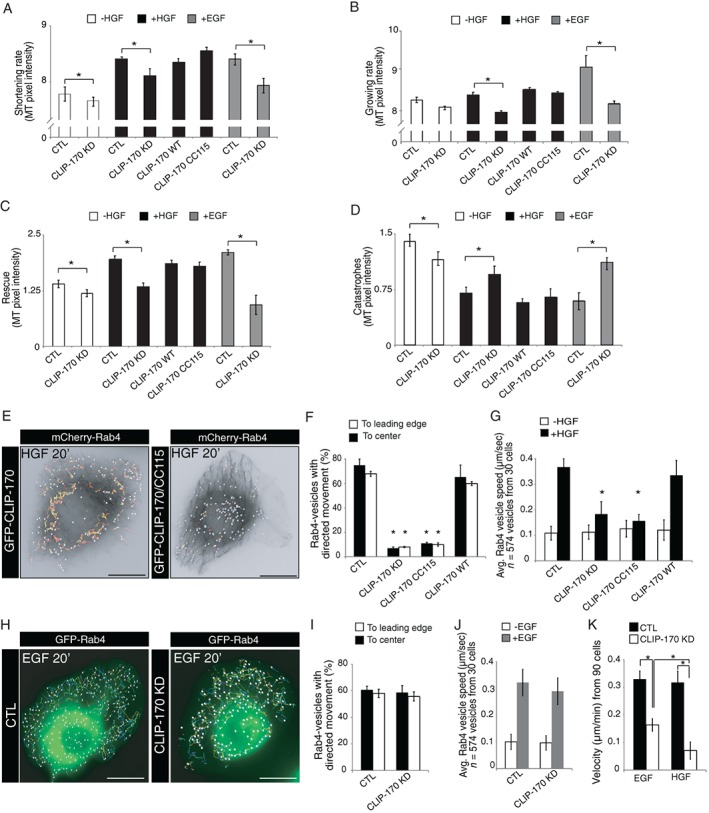
CLIP‐170 is not required for EGF‐induced Rab4‐positive vesicles movement. MT dynamics was analyzed by tracking plus‐ends of EGFP‐α‐tubulin‐labeled MTs over time after HGF (0.5 nM) or EGF (100 ng/mL) stimulation. (A) Shortening and (B) growing rate of MT, and (C) rescues and (D) MTs catastrophes were analyzed. (E) SKBr3 cells were co‐transfected with GFP‐Rab4 and CTL or CLIP‐170 siRNAs or GFP‐CLIP‐170 CC115 and were treated with HGF 0.5 nM, HGF (20 minutes). Individual Rab4‐positive vesicles were tracked over time. The percentage of directed movement (F) and speed of vesicles (G) were analyzed. (H) SKBr3 cells co‐transfected with GFP‐Rab4, and CTL or CLIP‐170 siRNAs, were treated with 100 ng/mL EGF (20 minutes). Individual Rab4‐positive vesicles were tracked over time. The percentage of directed movement (I) and speed of vesicles (J) were analyzed. (K) Cell velocity was determined by tracking cells after EGF addition in comparison with HGF stimulation. Scale bar = 10 μm. *P < 0.05

The role for CLIP‐170 in cell migration and its ability to regulate MT dynamics has been documented.^42^, ^46^ To assess the impact of CLIP‐170 depletion and the expression of GFP‐CLIP‐170 CC115 on HGF‐mediated MT dynamics, we examined the behavior of MTs in response to HGF and the EGF by live imaging in cells expressing EGFP‐α‐tubulin. Following CLIP‐170 depletion, a decrease in the frequency of MT rescues and an increase in the frequency of MT catastrophes were observed that was dependent upon HGF. The same defect was observed following EGF stimulation after CLIP‐170 KD, confirming that CLIP‐170 is an essential effector of HGF or EGF MT dynamics (Figure 5A‐D). Under HGF stimulation, the expression of CLIP‐170 WT or GFP‐CLIP‐170 CC115 is sufficient to restore the MT dynamics. Interestingly, the expression of GFP‐CLIP‐170 CC115 did not rescue the mobility of Rab4‐positive vesicles; their directionality and their speed were both altered in response to HGF (Figure 5E‐G). These results underline that the role of CLIP‐170 on the recycling of Met is independent of the defects in MT dynamics when CLIP‐170 is KD.

We also compared the localization and directionality of GFP‐Rab4‐positive vesicles under EGF stimulation to address the specific role of CLIP‐170 for HGF‐dependent Rab4‐positive vesicles mobility. In contrast to HGF, no significant difference in EGF‐mediated Rab4‐positive vesicle mobility was observed following CLIP‐170 depletion (Figure 5H‐J). However, CLIP‐170 depletion decreased cell velocity by 50% in response to EGF, but by 78% in response to HGF (Figure 5K). This supports an additional role of CLIP‐170 on Met recycling when compared to recycling of the EGFR.

### 2.5 CLIP‐170 binds Met RTK via the GAT domain of the GGA3 adaptor

**Figure 6:**
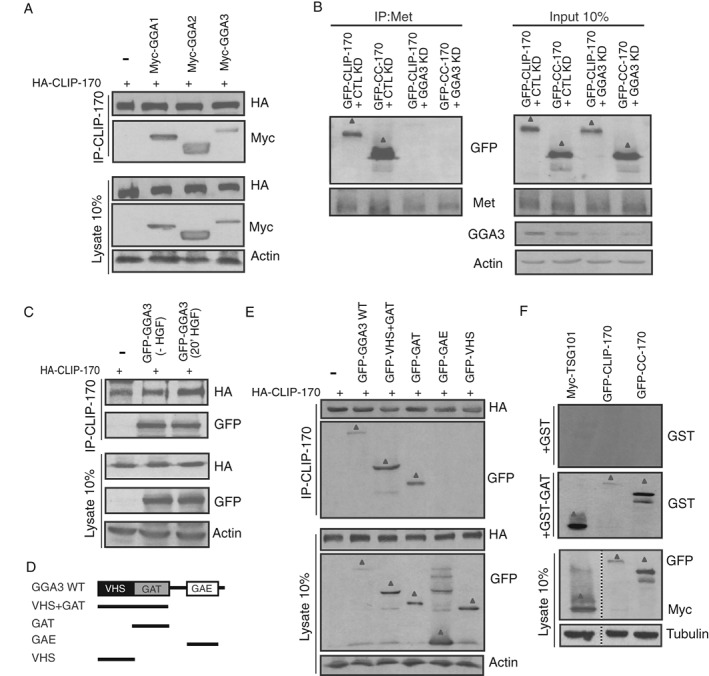
CLIP‐170 interacts directly with the GAT domain of the GGA3 protein. (A) HEK293 cells co‐transfected with HA‐CLIP‐170 and Myc‐GGA ‐1, ‐2 or, ‐3 proteins were subjected to IP with anti‐HA antibody and immunoblotted. (B) HeLa cells were co‐transfected with GFP‐CLIP‐170 or GFP‐CC‐170 and CTL or GGA3 siRNAs, stimulated with HGF 0.5 nM, HGF (20 minutes) and subjected to IP with Met or CTL IgG antibodies and immunoblotted. (C) HEK293 cells were co‐transfected with HA‐CLIP‐170 and GFP‐GGA3 and subjected to IP with anti‐HA antibody and immunoblotted. (D) Schematic diagram of GGA3 mutant constructs. (E) HEK293 cells co‐transfected with HA‐CLIP‐170 and GGA3 constructs shown in (D) were subjected to IP with anti‐HA antibody and immunoblotted. (F) HeLa cells transfected with GFP‐CLIP‐170, GFP‐CC‐170 or Myc‐TSG101 were subjected to IP with either anti‐GFP or Myc antibodies. Protein complexes were separated by SDS‐PAGE and transferred to nitrocellulose membrane. The membranes were incubated with fusion protein GST alone or GST‐GAT and immunoblotted. The bands of the proteins and mutants of interest are indicated by triangles

We previously reported that GGA3 protein is recruited to an activated Met RTK cargo complex that is present within the early tubular endosomal network via Crk and Arf6.^8^ As the inhibition of GGA3 by RNA interference had a similar impact to CLIP‐170 KD on Rab4‐positive vesicle mobility and cell migration upon Met stimulation, we tested whether GGA3 contributes to the ability of CLIP‐170 to associate with the Met RTK. To this end, we first investigated whether GGA1, 2 and 3 proteins could bind to CLIP‐170. Interestingly, CLIP‐170 coimmunoprecipitates with all three GGA proteins (Figures 6A and S4F). To establish if GGA3 modulates the formation of a Met CLIP‐170 complex, the ability of endogenous Met to coimmunoprecipitate with exogenously expressed GFP‐tagged CLIP‐170 WT or the CLIP‐170 CC domain was examined in the absence of GGA3. Compared to control cells, the Met association with CLIP‐170 WT or the CC mutant was decreased when GGA3 was depleted (Figure 6B). However, depletion of GGA1 or GGA2 did not affect this interaction (Figure S4G,H), supporting a specific role for GGA3 in the recruitment of CLIP‐170 via its CC domain to Met. CLIP‐170 coimmunoprecipitated with GGA3 in the absence of HGF, indicating that CLIP‐170 forms a constitutive complex with GGA3 (Figure 6C). Together, the data support that GGA3 is an interface for the molecular interaction between Met and CLIP‐170.

To further characterize the interaction of GGA3 with CLIP‐170, we performed structure‐function studies, which revealed that CLIP‐170 bound specifically to the GAT domain of GGA3 but not to the VHS and GAE domains (Figure 6D,E and S5A). Furthermore, to confirm whether this interaction is direct, we examined the ability of a GST‐fused GGA3 GAT domain to bind CLIP‐170 WT and the CC mutant. We also use a known GAT domain binding partner, TSG101 (tumor suppressor gene 101), which is an ESCRT component, as positive control in far‐western experiments (Figure 6F). As expected, the GAT domain of GGA3 bound to TSG101. Interestingly, we also observed an association with CLIP‐170 WT and with the CC mutant, revealing a previously undescribed direct interaction between CLIP‐170 and GGA3 (Figure 6F).

To confirm the role of the GGA3 GAT domain in the formation of a Met/CLIP‐170 complex, we measured the mobility of Rab4‐positive vesicles upon HGF stimulation in cells overexpressing GAT‐N194A, a GGA3 mutant that specifically uncouples it from interaction with Arf‐GTP proteins.^8^, ^47^ This mutant is also predicted to selectively disrupt the interaction with CLIP‐170 by competing with endogenous GGA3, while maintaining CLIP‐170 at MT plus‐ends. We observed that the GGA3 GAT‐N194A mutant coimmunoprecipitated with CLIP‐170 (Figure S5B,C), supporting that the GGA3/CLIP‐170 interaction is independent of Arf‐GTP activity. As expected, the net directionality of Rab4‐positive vesicle movement was decreased (Figure S5D) and the average speed of Rab4‐positive vesicles was reduced from 0.33 to 0.11 μm/s following GGA3 GAT‐N194A mutant overexpression (Figure S5E). Together, these results demonstrate that the GAT domain of GGA3 is necessary and sufficient for the association of Met‐positive vesicles with MTs via CLIP‐170, thereby relocalizing Met to the cell cortex.

## 3 DISCUSSION

**Figure 7:**
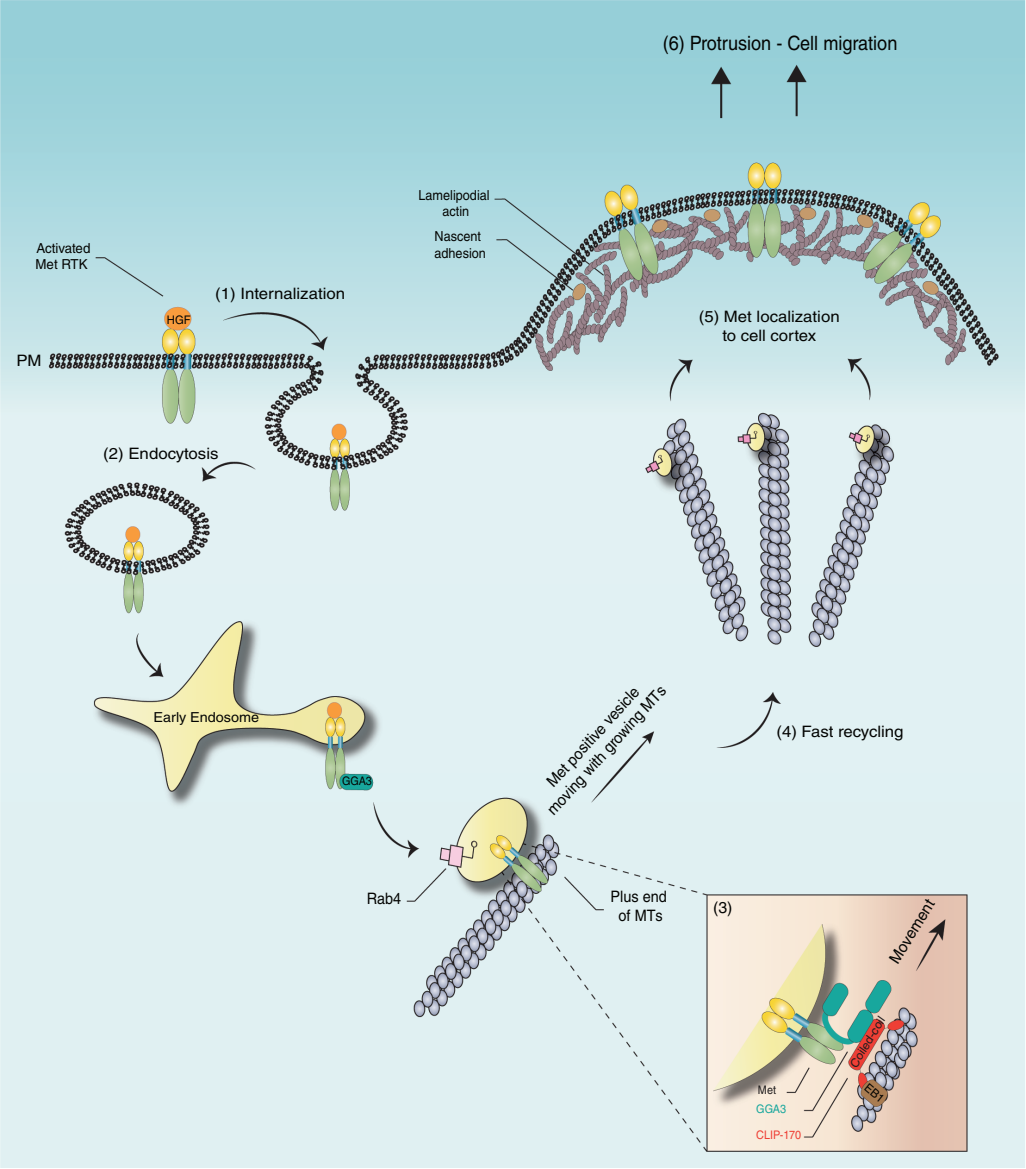
Hypothetical model of CLIP‐170 controlling Met RTK recycling in migrating cells. Upon HGF stimulation, Met is rapidly internalized (1) and undergoes endocytosis (2). The interaction of the C‐C domain of CLIP‐170 with the GAT domain of the adaptor GGA3 binds Met and leads to the anchoring of this complex at the growing plus‐ends of MTs (3). Met is sorted towards a direct (fast) pathway which depends on Rab4 GTPase (4) and recycled back to the cell surface by a microtubule‐based transport (5). Taken together, the efficient Met delivery allows the cells to dynamically modulate their adhesions and signaling, allowing efficient cell migration (6)

RTK recycling to spatially restricted PM microdomains in response to ligands remains poorly understood. In the present study, we describe a molecular mechanism whereby the +TIP, CLIP‐170, is required for recycling of Met RTK to the cell cortex and is linked to HGF‐dependent biological responses. Using structure‐function and rescue experiments with CLIP‐170 and associated proteins, we analyzed Met internalization and recycling. These multiple approaches yielded quantitative complementary data that support a model in which the formation of a Met/CLIP‐170 complex is mediated by the adapter protein GGA3 and promotes efficient interaction of Met/Rab4 recycling endosomes to the MT network. The enhanced localization of Met‐positive vesicles towards the MT plus‐ends following HGF stimulation modulates cell protrusion dynamics, initiating and maintaining a signaling microenvironment required for cell migration (Figure 7).

Depletion of other +TIP proteins, such as IQGAP1 or p150Glued, had no effect on Met localization to the cell cortex. These observations suggest that the impact of depleting these +TIPs on MT dynamics may be independent of Met RTK recycling. CLIP‐170 can localize to vesicular‐like structures^43^ and in vitro, CLIP‐170 promote interaction of endosome with MT,^48^ supporting the potential engagement of CLIP‐170 with Met‐positive vesicles. MTs also function in rapid recycling pathways involving the transferrin receptor,^23^ suggesting a model for MT involvement in the Met‐recycling pathway. Melanosomes require CLIP‐170‐dependent capture for their transport to the plus‐ends of MTs.^33^ CLIP‐170 also plays a role in the interaction of MTs with membrane organelles destined for movement to the MT minus‐ends by its ability to associate with the large subunit of the motor dynein activator dynactin, p150Glued trafficking of Met towards MT minus‐ends in the perinuclear compartment has been documented,^49^ although the mechanism of Met engagement with MTs has not been investigated. Here we report that expression of a CLIP‐170 mutant lacking the interaction domain with p150Glued (ΔC) was sufficient to rescue Met recycling and cell motility. Hence the MT minus‐end tracking capability of p150Glued is not involved in Met recycling to the cell cortex. This supports a distinct role for CLIP‐170 in promoting enhanced localization of Met to MT plus‐ends at the cell periphery as a requirement for Met recycling to the cell cortex.

The superfamily of kinesins is among the molecular motors involved in intracellular transport. Kinesins use MTs as a “rail” to transport cargo and to drive plus‐end‐directed anterograde transport.^50^ The kinesin KIF16B regulates endosome motility towards MT plus‐ends and regulates motility of EGFR‐positive endosomes enhancing EGFR recycling.^24^ It would be of interest to investigate whether a similar regulatory mechanism of Met plus‐end transfer could be orchestrated by a member of the kinesin superfamily after capture of Met‐positive vesicles by CLIP‐170. Using another stimulus such as EGF, we emphasized a specific role of CLIP‐170 on Met recycling. Moreover, because CLIP‐170 depletion did not affect the mobility of Rab4‐positive vesicles in response to EGF stimulation, we can postulate that this process is linked to Met rather than the Rab4 GTPase adaptor.

Structure‐function and rescue experiments revealed that removal of the head domain of CLIP‐170, which is required for interaction with MTs^51^ and other +TIP binding proteins such as EB1,^52^ did not alter binding of CLIP‐170 with the Met receptor. However, this CLIP‐170 mutant failed to rescue the enhanced mobility of Rab4‐positive vesicles containing Met to the cell cortex, as well as cell migration, in response to HGF. These results further establish CLIP‐170 as the link between Met‐Rab4 positive vesicles and MTs.

Given the high structural homology between GGA family proteins,^53^ we found that GGA3, as well as GGA1 and GGA2, interact with CLIP‐170. Our previous results showed that the association of GGA3 with Met RTK involved a constitutive interaction between GGA3 and the Crk adaptor protein.^8^ Consistent with this data, our results support a model where Met RTK‐positive vesicle is recruited to CLIP‐170 MT plus‐ends via an association with a GGA3‐Crk complex. The direct interaction of CLIP‐170 with GGA3 is mediated by the GAT domain of GGA3 and is independent of Arf‐GTP binding to GGA3. A GAT‐N194A mutant, which uncoupled GGA3 from interaction with Arf‐GTP proteins, maintained association with CLIP‐170. Consistent with this, the overexpression of the GAT‐N194A mutant competed with the GGA3‐CLIP‐170 interaction, but not with a GGA3 Arf6 interaction, and abrogated the directed movement of Rab4‐positive vesicles in response to HGF. Based on these findings, we provide new insights into GGA3 functions by linking the endosomal trafficking network and MT cytoskeleton transport via CLIP‐170.

Dynamic instability of MTs is controlled spatially and temporally by growing/shrinking phases and by catastrophes/rescue events,^25^, ^26^ which are modified by interaction with +TIPs.^54^ Hence, CLIP‐170 may spatially promote accessibility of Met/GGA3/CLIP‐170 Rab4‐positive endosomes to growing MT plus‐ends. The in vivo plus‐end tracking behavior^51^ as well as the in vitro property of CLIP‐170 linking endosomes and MTs^43^, ^48^ may also support the function of CLIP‐170 as a rescue factor stabilizing tracks containing Met/Rab4 endosomes. This interpretation is supported by a recent observation that EB1 is required for HGF‐induced epithelial cell protrusions in 3D cultures and trafficking of VAMP3‐positive endocytic vesicles into these protrusions.^37^ Our results provides a mechanistic understanding for this data where depletion of EB1 causes loss of CLIP‐170 from MT plus‐ends,^38^ resulting in decreased Met recycling to cell protrusions. However, the stabilization of MTs alone is insufficient for Met recycling because overexpression of EB3, which stabilizes MTs,^38^ failed to rescue Met recycling under conditions of CLIP‐170 depletion, highlighting a specific requirement for CLIP‐170 in Met recycling.

Our results support a model in which CLIP‐170 serves to promote association of Met/Rab4 positive recycling endosomes with dynamic MTs localized to the cell cortex of migrating cells via GGA3 (Figure 7). MT plus‐ends decorated with CLIP‐170 may be useful for searching and capturing relatively stationary cargoes loaded with Met, near cell periphery and perinuclear regions for an outward or inward transport. In some contexts, MT dynamics are mediated by the direct binding of membranous cargo to the growing or shrinking plus‐ends of MTs.^55^ Hence, engagement of Met with CLIP‐170 may provide a specific target in which to disrupt the HGF/Met signaling axis in cancers where Met signaling promotes enhanced migration and invasion and metastatic spread.

## 4 MATERIALS AND METHODS

### 4.1 Cell transfection and plasmid constructs

SKBr3 breast carcinoma cells, HEK293 and HeLa cells were grown in DMEM and 10% FBS. HeLa cell transfections were performed using Lipofectamine Plus according to manufacturer's instructions (Invitrogen). SKBr3 cells were transfected by Lipofectamine 2000 according to manufacturer's instructions (Invitrogen) or by nucleofection (Amaxa). SKBr3 cells were transfected with 2 μg siRNAs for GGA1 S103057663 (Qiagen), GGA2 S103190012 (Qiagen), GGA3,^8^ CLIP‐170 (nt 4472‐4495; within the 3′ untranslated region) or siGENOME SMARTpool M‐005294 (Dharmacon), P150Glued D‐012874‐01 (Dharmacon), IQGAP1 M‐004694‐02 (Dharmacon), EB1 sc‐35257 (Santa Cruz) and Allstars negative control siRNA (Qiagen). Transfections were also performed with the following plasmid constructs: GFP‐CLIP‐170 WT, RFP‐CLIP‐170 WT, GFP‐CLIP‐170‐ΔH and HA‐CLIP‐170‐ΔH (Kindly provided by Dr Yulia Komarova, University of Illinois); pEGFP‐C1‐CLIP‐170‐CC, pEGFP‐C1‐CLIP‐170‐ΔC, pEGFP‐C1‐CLIP‐170‐C1 and pGEX‐4T‐1‐CLIP‐170‐CC (Kindly provided by Dr Kozo Kaibuchi, Nagoya University); GFP‐GGA3 WT, GFP‐VHS + GAT, GFP‐GAT, GFP‐GAE and GFP‐VHS (Kindly provided by Dr Juan Bonifacino) and Myc‐TSG101 (Kindly provided by Dr Arnim Pause, McGill University). GFP‐Rab4 and GFP‐Rab11 (provided by Robert Lodge) were subcloned into a modified EGFP‐vector plasmid in which EGFP has been replaced by mCherry‐tag, using EcoRI and BamHI restriction sites, EGFP‐α‐tubulin (Clontech Laboratories, Inc.), GFP‐tagged C‐terminal EB3‐GFP and CLIP‐115 (Kindly provided by Dr Niels Galjart, Erasmus MC). The chimeric GFP‐CLIP‐170 CC115 construct was generated by PCR, digested using BglII, EcoRI and XhoI restriction sites and subcloned into the pEGFP‐C2 vector (Clontech Laboratories, Inc.).

GFP‐CLIP‐170 CC115 primer sequences are: 5′‐AAACTCGAGTCGGGCACGGCCTTGCAGGAGGC‐3′ and 5′‐AAAGAATTCTCAGTGCTTGTCCTCTTGTTTCTGAGCTTTGTCC‐3′. The construct GFP‐GGA3 GAT‐N194A was generated by site‐directed mutagenesis to introduce the mutation N194A in the GAT domain of GGA3 according to manufacturer's instructions (Stratagene).

GFP‐GGA3 GAT‐N194A primer sequences are: 5′‐GACCTGCAGGAGGCCGCCAAGCTCATCAAGTC‐3′ and 5′‐GACTTGATGAGCTTGGCGGCCTCCTGCAGGTC‐3′.

### 4.2 Live cell imaging

Cells were grown on collagen‐coated glass coverslips (35 mm, Ibidi GmbH) for 48 hours then stimulated for 20 minutes with HGF (0.5 nM) or EGF (100 ng/mL). Image were collected on a motorized stage equipped with an inverted microscope Axiovert 200 M (Carl Zeiss, Inc.), set at ×100 plan Apochromat NA 1.4 objective, with an AxioCam HRM digital camera (Carl Zeiss, Inc.). Cells were maintained within a chamber (Climabox, Carl Zeiss, Inc.) with 5% (v/v) CO_2_ in air at 37°C. The microscope was driven by the AxioVision LE software (Carl Zeiss, Inc.).

### 4.3 Analysis of MT dynamic instability

Cells were grown on collagen‐coated glass coverslips (35 mm, Ibidi GmbH) for 48 hours then stimulated for 20 minutes with HGF (0.5 nM) and observed with an inverted microscope Axiovert 200 M (Carl Zeiss, Inc.), and ×100 plan Apochromat NA 1.4 objective, AxioCam HRM (Carl Zeiss, Inc.). Thirty‐one images per cell were acquired at 4 seconds intervals using a digital camera (charge‐coupled device Coolsnap FX (Princeton Instruments); Coolsnap HQ (Roper Scientific). Plus‐ends of individual MTs were tracked in time using MetaMorph software (MDS Analytical Technologies). The number of catastrophes (transition from growth or pause to shortening) and rescues (transitions from shortening to pause or growth) was calculated as described previously^40^ from 30 MTs in three independent experiments. Means and (±) SE of the mean (SEM) are shown.

### 4.4 Three‐dimensional tracking of vesicles

For the movement of vesicles, the images in 3D (*X*, *Y*, *Z*) of GFP‐Rab4 expressing cells were acquired at 4 seconds intervals for 2 minutes, 10 sections and 0.5 μm spacing; parameters varied slightly in some experiments. Exposer time was fixed between 500 and 900 ms to avoid any over saturation. The images were acquired using an inverted microscope Axiovert 200 M (Carl Zeiss, Inc.), and ×100 plan Apochromat NA 1.4 objective. Cells were maintained within a chamber (Climabox, Carl Zeiss, Inc.) with 5% (v/v) CO_2_ in air at 37°C and 31 images per cell were acquired using a digital camera (charge‐coupled device); Coolsnap FX (Princeton Instruments); Coolsnap HQ (Roper Scientific); light Source, X‐Cite Metal Halide Lamp and fluorescence cube, FS 49002 (eGFP). The vesicle tracks were analyzed with Imaris software (Bitplane Inc.). After segmenting the cell volume by thresholds, the vesicles were detected with the spot detection function of Imaris. We used the Spots tracking algorithm with Brownian motion for automated cell localization and tracking, the Spots diameter was set to 0.5 μm and Background Subtraction function was added. For the speed quantification of Rab4‐positive vesicles, we used the Rab4 endosomes which were mobile, and vesicles that could be tracked for more than 100 seconds were evaluated. This was important to ensure that particles could be tracked sufficiently over a long duration to determine the overall directionality of their movement. Inclusion of shorter tracks did not alter the vesicle speed. To determine whether vesicles move to the cortex or center of cell, the center of mass of the cell volume was determined in Imaris, and the direction of the vector describing vesicle displacement relative to the cell center was determined.^56^

### 4.5 Motility assay

Live‐cell motility was analyzed as described previously^40^, ^57^ except that pictures were collected for 420 minutes at 5 minutes intervals. Lines of 1 pixel wide and 100 pixels long were used for kymograph analysis. Motility parameters including rates of migration, migration paths and kymographs were obtained from time‐lapse movies. Means of velocity and kymograph analysis of membrane protrusions were calculated using MetaMorph and Excel (Microsoft) software.

### 4.6 Immunoprecipitation and western blot analysis

Cells were harvested in RIPA lysis buffer (150 mM NaCl, 20 nM Tris HCl, 1 mM EDTA, 1 mM EGTA, 1% TritonX‐100, 1% deoxycholate, pH 7.4). All lysis buffers were supplemented with 1 mM phenylmethylsulfonyl fluoride (PMSF), 1 mM sodium vanadate, 1 mM sodium fluoride, 10 μg/mL aprotinin and 10 μg/mL leupeptin. Samples were resolved by SDS‐PAGE and transferred to nitrocellulose. Membranes were blocked with 5% bovine serum albumin and probed with appropriate antibodies, as described, and then with horseradish peroxidase‐conjugated secondary antibodies. All immunoblots were visualized by enhanced chemiluminescence (Amersham Biosciences).

For immunoprecipitations (IPs), lysates were incubated with antibody for 1 hour at 4°C with gentle rotation followed by 1‐hour incubation with protein A‐ or G‐Sepharose beads. Captured proteins were collected by washing three times in lysis buffers, eluted by boiling in SDS sample buffer and processed as above for western blotting. Antibodies were used against the following proteins: CLIP‐170, EB1, GGA1 (Santa Cruz Biotechnology, Inc.), Met antibody 147 (raised against a C‐terminal peptide of the human Met protein),^58^ Met pY1234/1235 (Cell Signaling), P150Glued, IQGAP1, GGA3 (BD Bioscience), Gab1 (Epitomics), Gab1 pY627 (Life technologies, Inc.), actin, α‐tubulin (Sigma‐Aldrich), Myc 9E10, HA.11, GST.B‐14 (Santa Cruz Biotechnology, Inc.) and GFP mouse (Roche, Inc.). Anti‐GGA2 was a kind gift from Dr Juan Bonifacino.

### 4.7 Far‐western blotting

HeLa cells were transfected with the indicated constructs, immunoprecipitated, separated by SDS‐PAGE and transferred to nitrocellulose membranes. Membranes were incubated with either GST or GST‐GGA3 GAT fusion proteins in lysis buffer (20 mM Hepes, pH 7.5, 120 mM NaCl, 2 mM EDTA, 10% glycerol, 1 mM PMSF, 10 mg/mL aprotinin and 10 mg/mL leupeptin) and bound GST‐fusion proteins were detected using an anti‐GST antibody.

### 4.8 Subcellular fractionation of PM/lamellipodia proteins

Proteins localized in lamellipodia were purified, as previously described.^40^, ^59^ Briefly, cells were plated on 3 μm porous polycarbonate Transwell membrane inserts (Costar) that were coated on the bottom side with 25 μg/mL rat tail collagen (Roche, Inc.). The lower chamber contained medium with 0.5 nM HGF. Cells were allowed to extend their lamellipodia through the pores. Cell bodies remaining on the upper surface were removed and the lamellipodia extending to the lower surface were recovered in lysis buffer.

### 4.9 Immunofluorescence microscopy

Cells grown on collagen‐coated coverslips were fixed in 4% formaldehyde at room temperature or 100% methanol at −20°C for MTs staining and permeabilized in 0.2% Triton X‐100 before the addition of antibodies. Antibodies used for IF were against the following: Met (AF276 from R&D Systems); α‐tubulin (Boehringer, Ingelheim), CLIP‐170, GFP mouse (Roche, Inc.). Secondary antibodies and Alexa Fluor 488, 546 and 633, and phalloidin were obtained from Molecular Probes. Images were recorded with a scanning confocal microscope (LSM 510 Meta laser; Carl Zeiss, Inc.) with a ×100 plan Apo 1.4 NA objective and driven by ZEN LE software (Carl Zeiss, Inc.).

### 4.10 3D‐structured illumination microscopy

The 3D‐structured illumination microscopy (3D‐SIM) on HeLa fixed cells was performed on a DeltaVision OMX V4 system (Applied Precision, a GE Healthcare Company), equipped with ×100 NA 1.4 immersion objective (Olympus, API Certified U‐PLANAPO); two Evolve EM‐CCD cameras (Photometrics); and 405, 488 and 568 nm diode lasers. The z‐sections were completed at a spacing of every 125 nm for a total raw data image count of 120 images per 1 μm sample z‐stack. Full super‐resolution of 0.3 μm thick image stacks with 40 × 40‐μm field of view (512 × 512 pixels unprocessed image size with 16 bits dynamic range) could be captured with a total acquisition time of 1 minute. For 3D‐SIM images, time series were compiled from single time points using Imaris software (Bitplane, Inc.) to create time‐lapse series. All raw images obtained in conventional mode were deconvolved using constrained algorithm (SoftWare X4, Applied Precision). This algorithm has been quantitatively verified to accurately represent the original 3D object.^60^

For clarity of display, small changes to brightness and contrast were performed on 3D reconstructions. Only linear changes were applied to the brightness and contrast of images. Nonlinear (gamma setting) changes to the images were not performed. The degree of colocalization, expressed as the Pearson's correlation coefficient (the proportion of all red intensities that have green components among all red intensities) was assessed by the colocalization analysis function of Imaris software (Bitplane). Results were logged into Excel (Microsoft Office) for analysis. All values are means ± SEM from three independent experiments.

### 4.11 Quantification of immunofluorescence

Quantification of colocalization was as described previously.^8^ Briefly, MetaMorph software was used for object‐based colocalization measurements. Images were smoothed with a 3 × 3 low‐pass filter, and endosomes were identified and counted using size estimates and intensity thresholds in each image set. Binary images were created for each set of endosomal spots and were combined pair‐wise to give only the “colocalized” spots. The minimum spot size was set to remove any small spots because of partial, and likely random, overlap of spots. Results were logged into Excel for analysis. Values for all analyses including colocalization and vesicle counting represent mean ± SEM.

### 4.12 Recycling assay

For biotinylation assays, cells were serum‐starved and pretreated with low levels of 10 nM lactacystin and 100 nM concanamycin inhibitors for 1 hour before chilling on ice and biotinylation for recycling assays, as described previously.^8^, ^61^ After biotinylation, cells were stimulated with 0.5 nM HGF at 37°C in the presence of inhibitors for 7 minutes to allow internalization. Cells were placed on ice, stripped with reducing reagent (100 mM sodium 2‐mercaptoethanesulfonic acid [MesNa] in 50 mM Tris‐HCl [pH 8.6], 100 mM NaCl, 1 mM EDTA and 0.2% BSA) to remove noninternalized biotinylated proteins, and then returned to 37°C. To determine percentage of internalized proteins that recycled, cells were returned to ice, subjected to a second reduction with MesNa prior to lysis, recovered with NeutrAvidin‐agarose beads, and immunoblotted for detection of Met levels. Percent recycled Met was determined by quantifying immunoblots (n = 3) using the LI‐COR Odyssey scanner and software (LI‐COR Biosciences). The cycloheximide, lactacystin, sodium 2‐mercaptoethanesulfonic acid and iodoacetamide were purchased from Sigma. EZ‐Link Sulfo‐NHS‐SS‐Biotin and NeutrAvidin were obtained from Pierce Chemicals. Lactacystin and concanamycin were from Calbiochem.

### 4.13 Immunofluorescence recycling assay

Quantification of colocalization was as described previously.^8^, ^21^ Briefly, cells grown on glass coverslips were pulsed with prewarmed (37°C) 0.5 nM HGF for 5 minutes, washed five times with Leibovitz‐15 medium containing 0.2% BSA at 4°C, and chased at 37°C for 20 minutes, then fixed and processed for IF. Cells were scored on the ratio of endosomal over PM staining of Met and reported as mean ± SEM (n = 4). MetaMorph software was used for object‐based colocalization measurements as described above (see Section 4.11). In each experiment, a minimum of 20 fields was scored.

### 4.14 Quantification of Met aggregated

To determine the percentage of cells with aggregated (A), partially aggregated (PA) or dispersed (D) Met, GFP alone or EB3‐GFP transfected cells were stimulated with 0.5 nM HGF for 20 minutes and fixed with formaldehyde. The Met‐positive vesicles “at the MTs plus‐ends” have been scored at the vicinity of +end, stained with EB3‐GFP. The number of cells with aggregated, partially aggregated or dispersed Met were determined as previously described.^33^ Briefly, cells in each category have been counted under a scanning confocal microscope (LSM 510 Meta laser; Carl Zeiss, Inc.) with a ×100 plan Apo 1.4 NA objective and driven by ZEN LE software (Carl Zeiss, Inc.). In each experiment, a minimum of 20 fields was scored. See Figure S3D for representative images of each cell types.

### 4.15 Statistics

All results are presented as mean ± SEM based on data averaged across multiple independent experiments. The n value of an experiment represents experiments performed on different days unless otherwise noted. To assign significance, results were compared with control experiments by means of an unpaired *t* test using Excel (Microsoft Office).

## Supporting information


**Figure S1** **.** MT cytoskeleton and CLIP‐170 are required for Met‐positive early endosome dynamics upon HGF stimulation.
**Figure S2.** Dissociation of CLIP‐170 from MTs abrogates Met recycling and signaling.
**Figure S3.** GGA3 depletion affects Rab4‐positive vesicles mobility.
**Figure S4.** GGA3, but not GGA1 or GGA2 is required for Met/CLIP‐170 interaction.
**Figure S5.** CLIP‐170 bound to the GAT domain of GGA3. Arf6 GTPase is not required for CLIP‐170 interaction with GGA3.

**Movie S1**

**Editorial Process**
