# Supplementary material for: CLIP‐170 spatially modulates receptor tyrosine kinase recycling to coordinate cell migration
Source: Traffic. 2019 Jan 15;20(3):187–201. doi: 10.1111/tra.12629 (PMC6519375; doi:10.1111/tra.12629)
Supplement: Supplementary file 1 — Figure S1 . MT cytoskeleton and CLIP‐170 are required for Met‐positive early endosome dynamics upon HGF stimulation. Figure S2. Dissociation of CLIP‐170 from MTs abrogates Met recycling and signaling. Figure S3. GGA3 depletion affects Rab4‐positive vesicles mobility. Figure S4. GGA3, but not GGA1 or GGA2 is required for Met/CLIP‐170 interaction. Figure S5. CLIP‐170 bound to the GAT domain of GGA3. Arf6 GTPase is not required for CLIP‐170 interaction with GGA3. [file TRA-20-187-s001.pdf]

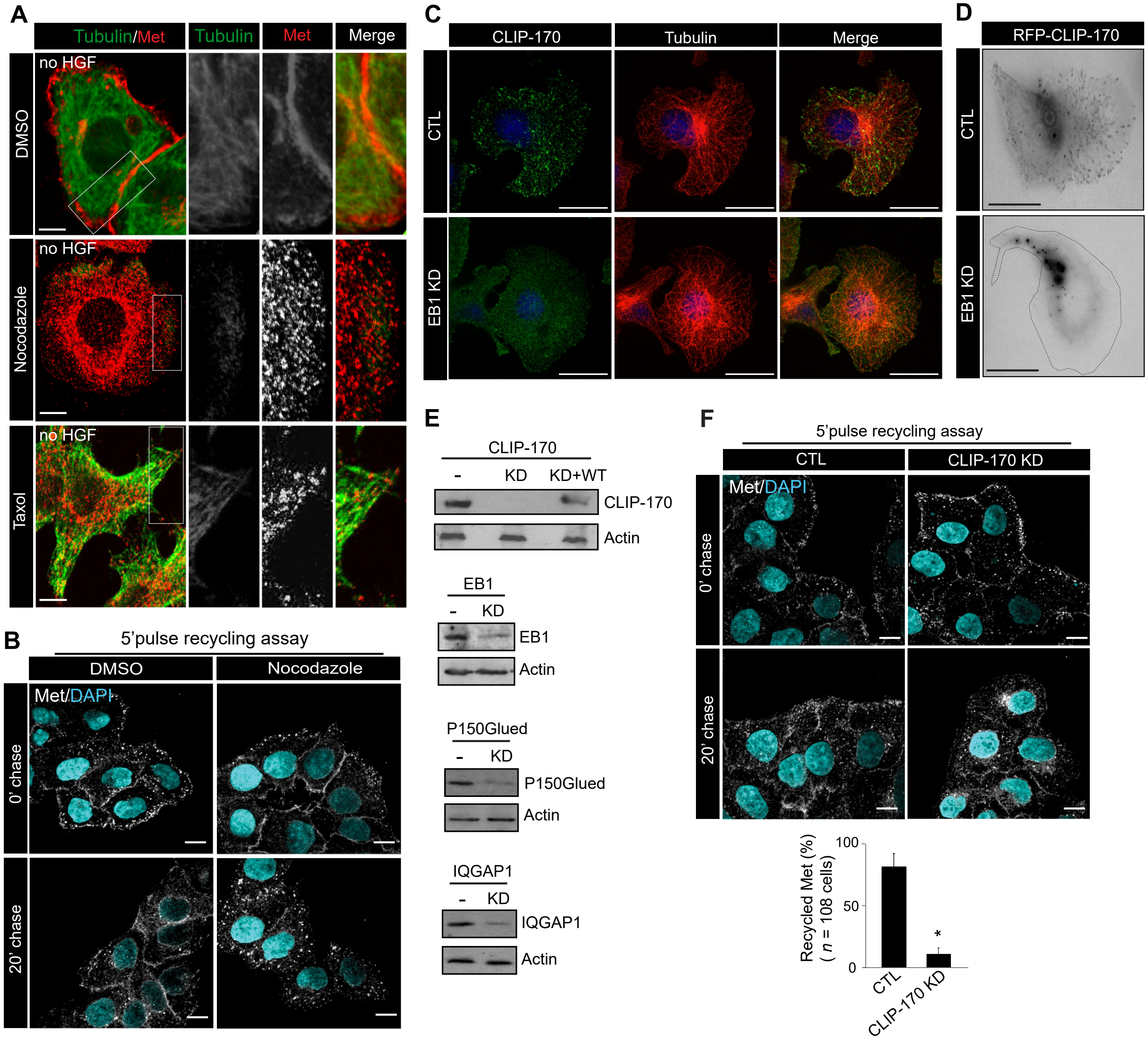

Figure S1. MT cytoskeleton and CLIP-170 are required for Met-positive early endosome dynamics upon HGF stimulation. (A) Representative IF staining of HeLa cells treated with DMSO or nocodazole (20 μM) for 10 minutes or taxol (0.1 μM) for 20 minutes and stained for Met (red) and tubulin (green). Inset shows higher magnification. (B) HeLa cells pre-treated with DMSO or nocodazole, pulsed for 5 minutes with HGF (0.5 nM) at 37ºC to allow internalization of Met receptors into early endosomes, then rapidly washed at 4ºC to remove unbound ligand and chased for 20 minutes to allow recycling. Representative images are shown. (C) Representative IF staining of endogenous CLIP-170 in HeLa cells in the presence or absence of EB1 after HGF stimulation (0.5 nM, 20 minutes). (D) Still images from time-lapse movies showing SKBr3 cells expressing RFP-CLIP-170 in the presence or absence of EB1 after HGF stimulation (0.5 nM, 20 minutes). EB1 KD blocks CLIP-170 association with MTs. Insets show enlargement from the cell cortex. (E) Western blot showing KD of CLIP-170, EB1, IQGAP1, P150Glued by siRNAs and rescue of CLIP-170. (F) Bottom, recycling assay in HeLa cells transfected with CTL or CLIP-170 siRNAs. Right, percentage of recycled Met from IF images was determined and quantified. Scale bar = 10μm. * $p < 0.05$.

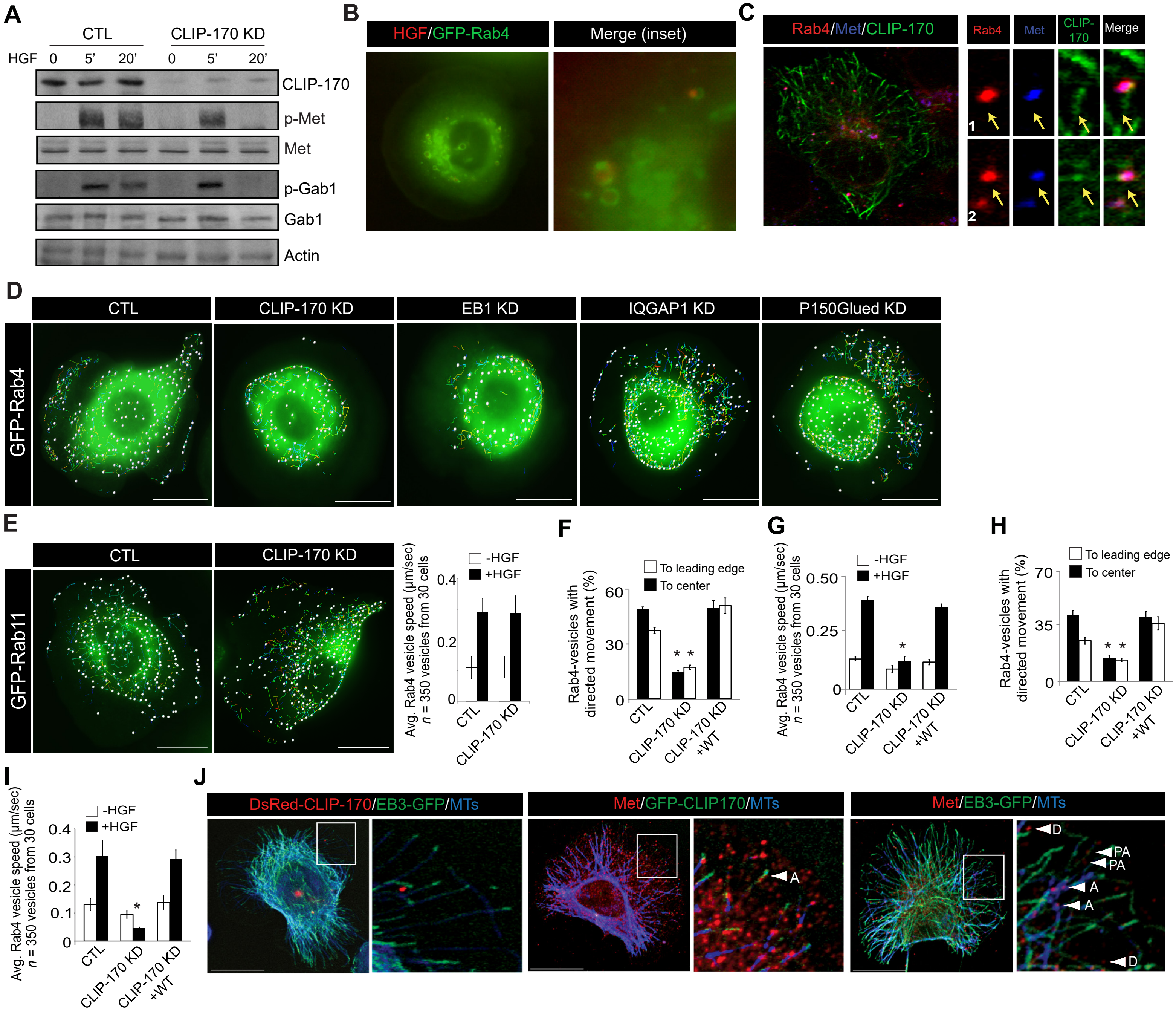

Figure S2. Dissociation of CLIP-170 from MTs abrogates Met recycling and signaling. (A) Western blot of p-Met and p-Gab1 level after 5 minutes and 20 minutes HGF (0.5 nM) stimulation in HeLa cells rescued with CLIP-170 (see panel S1E). (B) Still image from time-lapse series showing SKBr3 cells expressing GFP-Rab4 stimulated for 20 minutes with fluorescently labeled HGF (HGF-Alexa555). Insets show enlarged endosomes (Movie S1). (C) HeLa cells co-expressing GFP-CLIP-170 and mCherry-Rab4, treated with HGF 0.5 nM, HGF (20 minutes), fixed and immunostained for Met (blue). Representative images at high magnification are shown. Arrows indicate colocalization of these three proteins. (D) SKBr3 cells co-transfected with GFP-Rab4 and CTL, CLIP-170, EB1, IQGAP1, or P150Glued siRNAs were treated with HGF. Individual Rab4-positive vesicles were tracked over time. (E) Left, cells co-expressing GFP-Rab11 and CTL or CLIP-170 siRNAs were analyzed after 20 minutes stimulation with 0.5 nM HGF. Right, quantification of speed of Rab11 vesicles after depletion of CLIP-170. (F-I) SKBr3 cells co-transfected with GFP-Rab4 and CTL or CLIP-170 siRNAs +/- CLIP-170-WT, were treated with 0.5 nM HGF for 20 minutes. Individual Rab4-positive vesicles were tracked over time. The percentage of directed movement (F) and speed of vesicles (G) were analysed using one plan imaging. The percentage of directed movement (H) and speed of total vesicles (I) were quantified using spinning disc microscopy. (J) Left, EB3-GFP expression affects the binding of CLIP-170 (red) to MT plus ends in HeLa cells. Middle, representative image of GFP-CLIP-170 transfected HeLa cells with aggregated (''A" arrowheads) Met-positive vesicles at the MTs plus-ends. Right, representative image of EB3-GFP transfected HeLa cells with aggregated (''A" arrowheads), partially aggregated (''PA" arrowheads) or completely dispersed ("D" arrowheads) Met-positive vesicles at the MTs plus-ends. Scale bar = 10 μm. * $p < 0.05$.

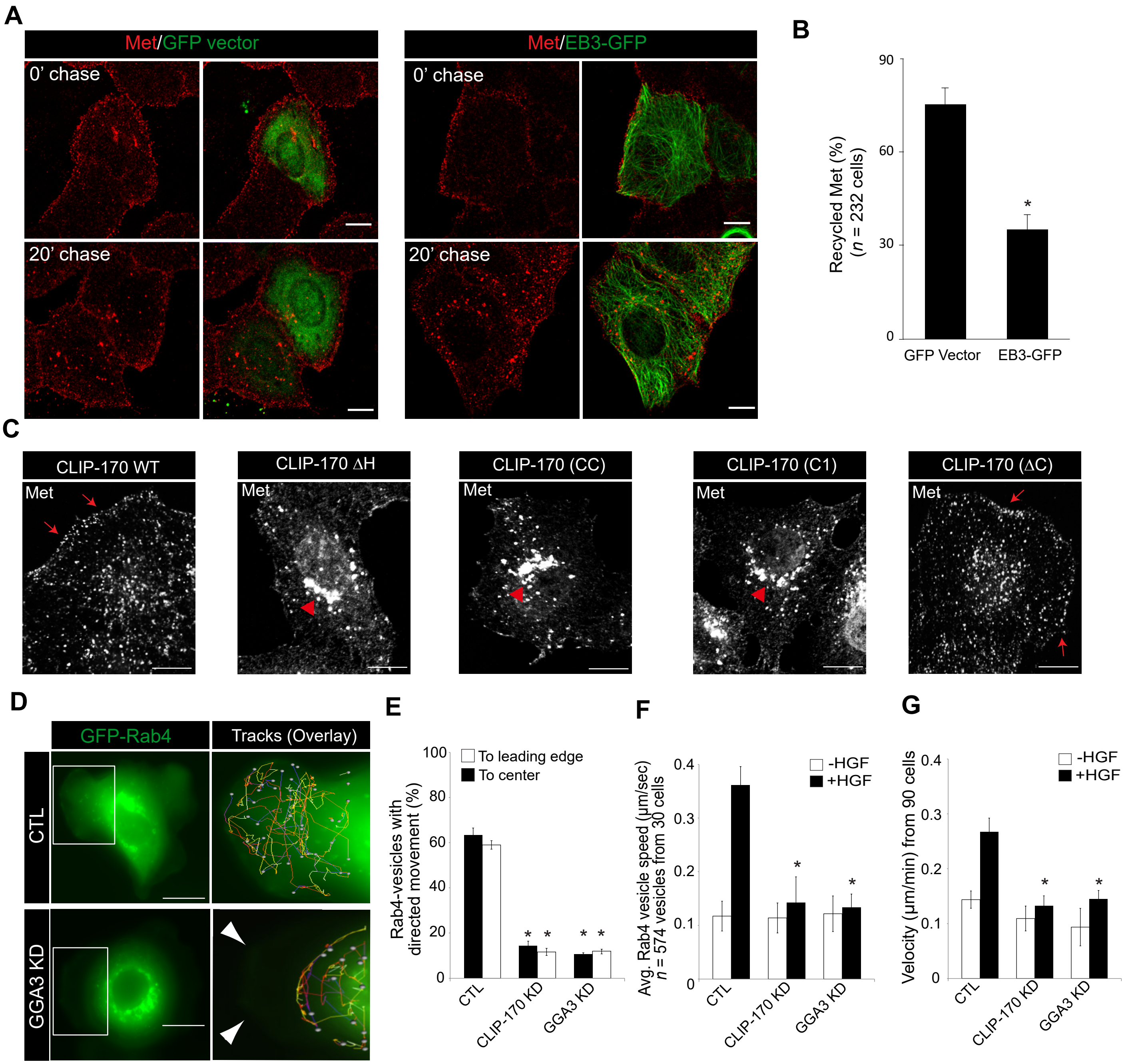

Figure S3. GGA3 depletion affects Rab4-positive vesicles mobility. (A) HeLa cells expressing GFP-vector or EB3-GFP were pulsed for 5 minutes with HGF (0.5 nM) at 37°C, washed at 4°C and chased for 20 minutes to follow Met recycling. (B) Percentage of recycled Met from IF images was determined and quantified. (C) Images of HeLa cells co-transfected with CLIP-170 siRNA and the indicated CLIP-170 constructs (WT and mutants; ΔH, ΔC, CC, C1), stimulated with HGF 0.5 nM for 20 minutes and stained with anti-Met antibody. Arrows indicate Met localization at the cell cortex, arrowheads indicate Met localization in the perinuclear region. (D) SKBr3 cells co-transfected with GFP-Rab4 and CTL or GGA3 siRNAs were treated with HGF 0.5 nM for 20 minutes. Insets show enlargement from the cell cortex. Arrowheads indicate the PM. Individual Rab4-positive vesicles were tracked over time (2 minutes) after 20 minutes stimulation with HGF 0.5 nM. The percentage of directed movement of vesicles (E) and the speed of Rab4-positive vesicles (F) were analyzed. (G) Cell velocity was determined by tracking cells every 5 minutes for 420 minutes. Ninety cells were tracked for CTL, CLIP-170 KD and GGA3 KD cells. Scale bar = 10μm.

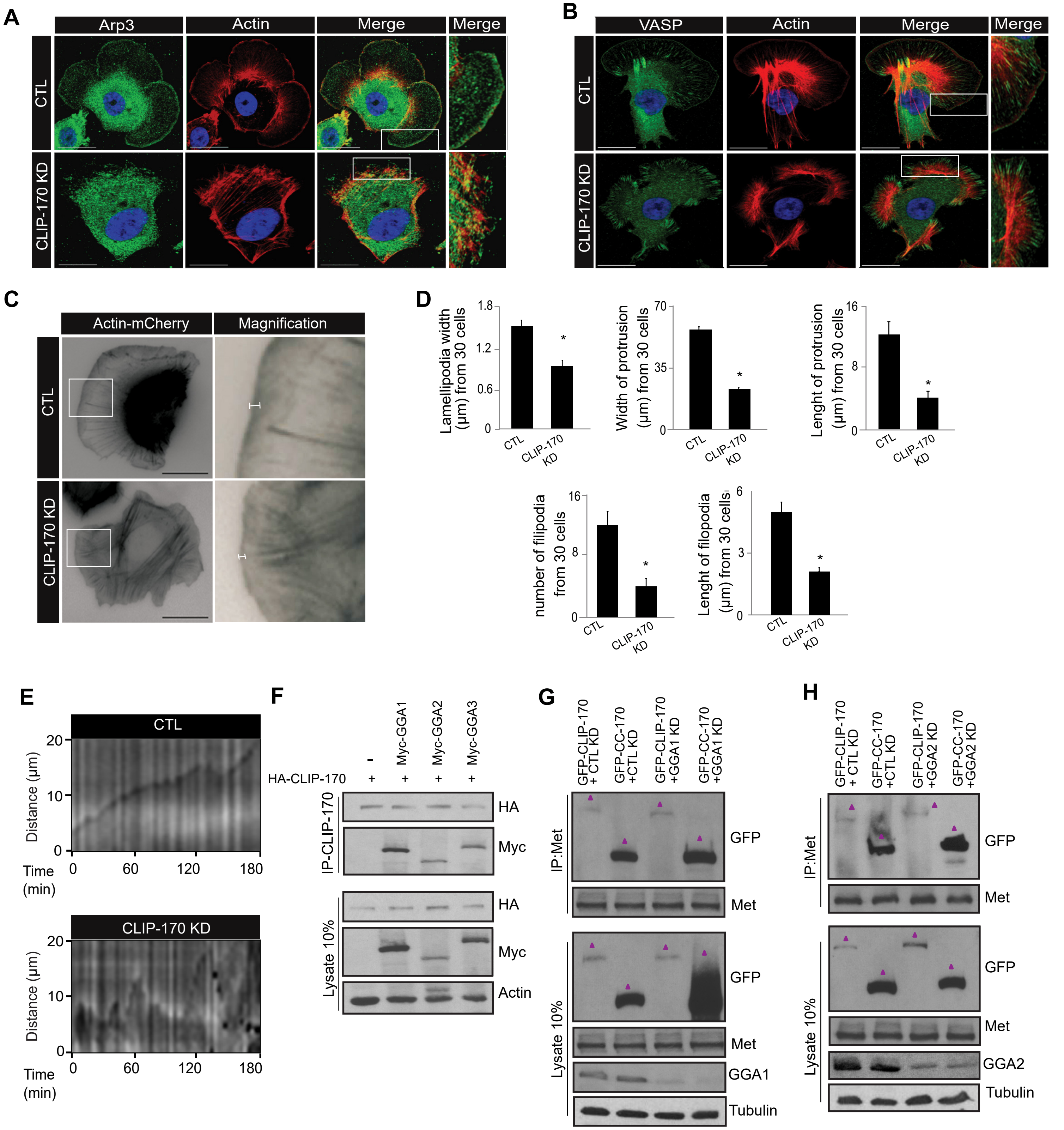

Figure S4. GGA3, but not GGA1 or GGA2 is required for Met/CLIP-170 interaction. (A-B) SKBr3 cells were treated with HGF (0.5 nM) for 20 minutes and immunofluorescence of actin and endogenous Arp3 (A) or endogenous VASP (B) were performed. (C) SKBr3 cells were co-transfected with Actin-mCherry and CTL or CLIP-170 siRNAs and were treated with HGF (0.5 nM) for 20 minutes. (D) Quantification of lamellipodia, protrusions and filopodia in CTL and CLIP-170 KD cells. (E) Kymograph analysis of PM protrusions in HeLa cells transfected with CTL or CLIP-170 siRNAs following HGF (0.5 nM) stimulation for 20 minutes. (F) HEK293 cells co-transfected with HA-CLIP-170 and Myc-GGA -1, -2, or -3 proteins were stimulated with HGF and subjected to immunoprecipitation (IP) with anti-HA antibody and immunoblotted. HeLa cells were co-transfected with GFP-CLIP-170 and CTL, GGA1 (G), or GGA2 siRNAs (H), stimulated with HGF and subjected to IP with Met or CTL IgG antibodies and immunoblotted as indicated. The bands of the proteins and mutants of interest are indicated by triangles. * $p < 0.05$.

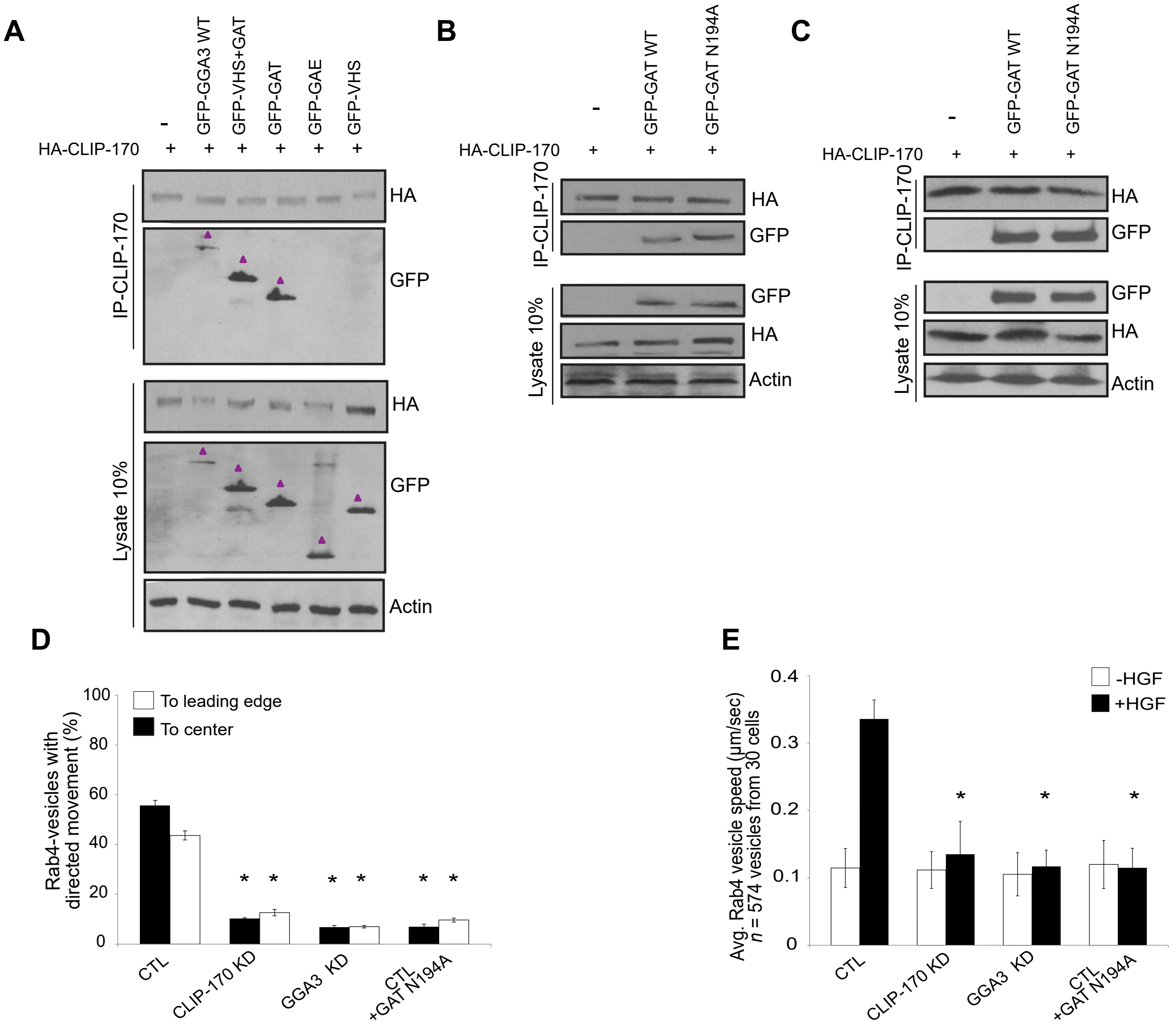

Figure S5. CLIP-170 bound to the GAT domain of GGA3. Arf6 GTPase is not required for CLIP-170 interaction with GGA3. (A) HEK293 cells co-transfected with HA-CLIP-170 and GGA3 constructs shown in Fig. 6D were stimulated with HGF 0.5 nM for 20 minutes and subjected to immunoprecipitation (IP) with anti-HA antibody and immunoblotted as indicated. (B) HEK293 cells co-transfected with HA-CLIP-170 and GGA3 GAT WT or GGA3 GAT N194A mutants were stimulated with HGF (B) or without (C) and subjected to IP with anti-HA antibody and immunoblotted as indicated. The percentage of directed movement (D) and speed of total vesicles (E) were quantified. The bands of the proteins and mutants of interest are indicated by triangles. * $p < 0.05$.
