# Supplementary material for: CLIP‐170 spatially modulates receptor tyrosine kinase recycling to coordinate cell migration
Source: Traffic. 2019 Jan 15;20(3):187–201. doi: 10.1111/tra.12629 (PMC6519375; doi:10.1111/tra.12629)
Supplement: Supplementary file 3 — Editorial Process [file TRA-20-187-s003.pdf]

Title: **CLIP-170 spatially modulates receptor tyrosine kinase recycling to coordinate cell migration**

Authors: Kossay Zaoui, Stephanie Duhamel, Christine Anna Parachoniak and Morag Park

Article Type: Original Research

Monitoring Editor Trina Schroer
Date Submitted 23 May 2018
Date for Decision 1 3 July 2018
Date Resubmitted 1 November 2018
Date for Decision 2 27 November 2018
Accepted 5 December 2018

Decision and Reviews

Dear Dr. Park,

Thank you for submitting your manuscript "CLIP-170 spatially modulates receptor tyrosine kinase recycling to coordinate cell migration" for consideration for publication in Traffic. I asked two colleagues who are experts in the field to review the paper and their verbatim comments are appended below. The referees share the view that the work presented in this paper will be of interest to the readers of Traffic. However, they have each raised concerns that you will need to address before the paper can be reconsidered. Many of these concerns can be addressed by revision of the text, but some additional data will also be required. The referees have very clearly outlined their concerns so there is no need for me to reiterate these here.

Although I cannot accept your manuscript for publication at this point, I believe that you will be able to address the referees' concerns and I look forward to receiving your revised manuscript. To expedite handling when you resubmit please be sure to include a response outlining how you have addressed each of the referees' concerns.

Sincerely,

Trina A. Schroer, Ph.D.
Co-Editor

____

Referee's Comments to the Authors

Referee: 1

Comments to the Author
In this work, the authors identify a new interaction between CLIP170, GGA3 and the Met receptor following stimulation of cells with HGF, and demonstrate that this is crucial for recycling the receptor, and for cell migration. This is convincingly shown using a range of complementary techniques. Importantly, they show that other depletion of other microtubule +TIPs does not affect this trafficking, and that defects in recycling after CLIP170 depletion are not due to alterations in MT dynamics (although those do occur). While much of the data are convincing, there are a number of technical issues that need to be addressed, particularly concerning the live cell imaging.

1. The authors have spent a great deal of effort analysing the effects of various manipulations on GFP-Rab4 vesicle movement, since they have previously shown that a significant proportion of the Met receptor resides in these organelles 20 mins after HGF uptake. My understanding of the methods used is that a complete z-stack is taken every

4 seconds, meaning that there is a time interval of 4 sec between equivalent slices. Furthermore, it is also not clear whether the tracking software will be able to track all GFP-Rab4 structures, regardless of size. This raises the real possibility that only the slower moving, larger Rab4 vesicles are tracked by the software, since faster ones move too far between frames and smaller ones could be missed altogether. In that case, the analysis is performed on only a subset of motile events. Although this doesn't detract from the observations that the Rab4 motility they measure is greatly affected by CLIP170 depletion (etc), it is important to know how complete this data set is. Their previous work used much faster imaging of single planes, rather than slow imaging in 3D, and could be used to assess the full range of effects on movements in one condition: plus/minus CLIP170.

2. More detail should be provided on the imaging system used for live cell imaging, in particular the illumination source and level of photodamage. Another concern is how well controlled the level of GFP-Rab4 expression is between movies, as they vary considerably as presented. Rab overexpression can give major artefacts, including enlarged endosomes and altered motility, so it is important to know how over-expressed it is here. In addition, it is very hard to make out the Rab4 labelling, as each image is shown in green/black rather than greyscale, and always have the tracking data superimposed. The authors should also show controls to demonstrate that microtubule organisation and cell morphology is not affected by their manipulations, as many of the depleted cells have a "fried egg" appearance in the Rab4 images.

3. On p3 is it stated that "Treatment of cells with nocodazole or taxol abrogated HGF-dependent Met recycling from endosomes back to the leading edge". However, from the methods, it is not clear at all how this IF-based recycling assay is quantitated. How is the intensity of the plasma membrane staining distinguished from the endosomal staining, and how is it determined? How is the ratio generated? Or is it simply the ratio of peripheral staining vs. central staining? If so, say that, and explain how the two regions are differentiated, and if the area sampled (i.e. number of pixels) is taken into account. By the way, the text used to describe the analysis is identical to their previous paper (Parachoniak et al., 2011). The IF recycling assay is backed up by a cell fractionation approach (Fig. 1D). However, a single blot is shown, without any quantitation. Can the authors quantitate this change in 3 independent experiments?

4. In the same experiment, Fig. S1A shows that the level of Met drops dramatically in taxol-treated cells. What is going on?

5. In Fig. S1B, it would be much better to show the effect of EB1 KD on native CLIP170 distribution, not on exogenously-expressed RFP-CLIP170. The KD image shown has a large CLIP170 aggregate in the centre, but this could be an over-expression artefact.

6. The authors state on page 4 that they are expressing a dominant negative EB3-GFP, but then contradict this in the text and Fig 2 legend to imply that it inhibits CLIP170 binding simply because it is over-expressed. Please clarify which is correct. On p4 and in Fig 2G "the percentage of cells with aggregated…Met-positive vesicles at the MTs plus ends" is assessed. What does this mean? Are they only scored in the vicinity of +ends (and if so, how are the +ends identified), or is this just inaccurate writing? There is no information in the methods on what has been done. To back up this scoring, representative images of each category should be included as supplemental data.

7. In Fig. 3D it appears that the CLIP170-deltaH interacts better with Met than full length CLIP170. Is that correct? Does that hint at regulation of the interaction via changes in CLIP170 conformation?

8. In the discussion on p 10 the authors state that "The enhanced localization of Met-positive vesicles towards the MT plus-ends following HGF stimulation, increases the stability of MT growth to lamellipodia…". They have not shown this at all, and the two statements are not necessarily linked. In the final paragraph of the discussion they suggest that Met-vesicles bound to CLIP170 are transported by plus-end motors. Do the authors mean that they are first transported with the kinesin, then bind to the +end via CLIP170? The alternative wouldn't work, since if they are already at +TIPs via CLIP170 binding, then there is no MT for a kinesin to move along. It would seem more likely that the +end movement

Minor points
The title of Fig. S5 is misleading, as the involvement of Arf6 GTPase has not been directly tested.

On p 5, it would be better to write "…levels of Met returning to the surface to 15% compared to 42%..." (rather than "by 15%") and also later in the same sentence "…to 7%" rather than "by 7%".

On p6, Fig 3G should read Fig 3F, and 3H should be 3G.

In Fig. 4, it is stated that "…the data represent the trajectories of ten cells". Presumably this is 10 cells in 3 repeats, meaning 30 cells (otherwise the numbers in the graphs don't make sense).

The legend title for Fig. 5 is missing a verb. Do they authors mean "…Rab4-positive vesicle movement"?

The authors should refer to the reported interaction between cytoplasmic dynein and Rab4, in Bielli et al. (2001). Biochemical and Biophysical Research Communications, 281(5), 1141–1153.

Referee: 2

Comments to the Author
MS ID: TRA-18-0759
"CLIP-170 spatially modulates receptor tyrosine kinase recycling to coordinate cell migration."
Kossay Zaoui, Stephanie Duhamel, Christine Anna Parachoniak, and Morag Park

In this manuscript, the authors describe that recycling of the receptor ryrosine kinase hepatocyte growth factor receptor (also known as c-Met) occurs in a microtubule-dependent fashion, and in association with a prominent +tip targeting protein of microtubules known as CLIP-170. Initially after HGF treatment of cells, Met is internalized to end up in Rab4-positive endosomes, which together with GGA3 (Golgi-localized γ-ear-containing Arf-binding protein 3), are recycled back to the plasma membrane. And all this then, the authors propose, promotes HGF-induced signaling to lamellipodia formation and HGF-triggered cell migration.
The manuscript contains quite a lot of data, and the results of most of the experiments look pretty straight forward and reasonable, but I have one major objection with the overall conclusion of the study, which is as follows: The authors imply in their conclusions and in the interpretation of their results in general that it is important for cells to position their receptors (in this case c-Met receptor) at what they call the "leading edge", so at the plasma membrane tips of in this case lamellipodial protrusion and/or membrane ruffles. This view can be readily deduced from the summary model on page 34 of the submitted manuscript (step 5), in which Met-receptor dimers localize at the tips of membrane protrusions, quasi ahead of the lamellipodial actin network and nascent adhesions. In fact, this is by no means realistic, and indeed the data don't show this either, and in addition, I have never seen clear-cut data from any other lab that has shown such a localization for RTKs, such as Met, EGFR or PDGFR. Instead, it was previously shown by TIRF microscopy, for instance, that EGFR can accumulate at clathrin-coated structures, and its endocytosis coincide with clathrin-mediated endocytosis (see e.g. Benesch et al., J Cell Sci 118 (Pt14):3103-15), so can occur scattered over the entire cell surface.
So I would agree with the authors that Met trafficking on Rab4-positive vesicles may occur to the plasma membrane, but I would refrain from stating that this occurs in a directional fashion towards the leading edge, and that this then is relevant for accumulation of the receptor at the leading edge membrane of lamellipodial protrusions, as no evidence for this is provided!

Specific Critique:

1) In Figure 1, we are looking at the distribution of microtubules and Met by indirect IF, but no evidence is provided that the spots we are looking (in red) actually correspond to Met-containing structures. Can the authors provide a control experiment to prove that the antibody staining is specific for Met, such as showing that in Met knockdown or -KO cells, no such staining is observed?

2) In all Figures, the authors jump forth and back between cell types, so HeLa-cells in Figure 1, SKBr3 in Figure 2, HeLa again in Figure 3 and so on. I don't know which cell type was used in 4, Figure 5 is SKBr3 again, HEK293 in Figure 6, and so on and so forth also throughout the Supplement. No explanation is provided why the authors have chosen a specific cell type for a given experiment, so this looks a bit like a wild mixture of data that has been assembled together.
I would recommend the authors to clearly state in the manuscript why a given cell type was chosen and is shown in a given Figure to make this clear.

3) As far as I understand, much of the conclusions are currently building on RNAi-mediated knockdown of CLIP170 and other factors, but it is not clear if the authors have convincingly shown by Western that the rescue construct (of CLIP170) is re-expressed; I could only find reduction of CLIP170 expression in the knockdown. I guess Figure S2A shows the reduction of CLIP170 expression in the knockdown situation, but the rescue could not be found. Moreover, it is a bit dangerous to solely rely on just one type of siRNA, as normally, at least two independent siRNAs are used for documenting the consequences of knockdown of a given factor. I would recommend repeating experiments with a second, independent siRNA sequence, and/or confirm the functional data with an alternative approach of interference with gene function, such as CRISPR/Cas9.

4) The protrusion analysis shown in Supplementary Figure S4 does not make much sense to me. The kymography is OK, in principle, but the contrast in the phase-contrast images is very poor. Most importantly, though, the “width” and “length” analysis (see Figure S4B) does not have anything to do with a reasonable classification of lamellipodial protrusions, as the authors are simply measuring the distance between periphery and nucleus (and for the width a distance orthogonal to that), without any distinction between different subcellular areas, such as lamellipodium versus lamella further proximal to the lamellipodium etc. The type of analysis provided here can perhaps give some indirect information on how large the area is the cells are adopting (i.-e. cell spreading efficiency, although there is much more elegant ways to do this), but it can by no means provide any information on protrusion parameters. For the latter, the authors would have to perform actin filament stainings, e.g. using fluorescent phalloidin, and then analyze the dimension of lamellipodia and/or other types of protrusions at the cell periphery. With the poor quality phase contrast images shown here, no clear classification of protrusions is possible, and hence very limited information can be obtained from such analyses.

5) In Figure 5, the authors also describe effects of CLIP170 knockdown on microtubule dynamics, such as growth or shortening rate and rescue or catastrophy frequencies. First, the table shown in Figure 5A indicates that the differences with many of these parameters are quite small.
More importantly though, the authors also claim that the differences observed were dependent on HGF, which would be quite astonishing to me…(see middle of 2nd para on the left)!
However, I can’t discern where evidence for this is shown, as all conditions in Table 1 are either “with HGF” or “with EGF”, but no data for the absence of those growth factors are shown, correct?

Minor comments:

6) In Figure 2J, I guess the authors meant to say that after 13 minutes of HGF treatment, levels of recycled Met were reduced to 7% (not “by 7%”) as compared to 55% in the control!

7) In Figure 3D, Lysate is mis-labelled as “Lystae 10%!

8) At the end of the discussion, the authors propose that Met-positive vesicles that are bound to CLIP-170 are transported by plus-end-directed kinesins, although in my view, CLIP170-mediated tracking on microtubule tips alone might be sufficient for the observations made in this manuscript. I feel that without any evidence for the involvement of kinesins, such statements should clearly be avoided!

Author Rebuttal

Dear Dr. Trina A. Schroer,

I am pleased to re-submit a revised version of our manuscript, originally TRA-18-0759, entitled **“**CLIP-170 spatially modulates receptor tyrosine kinase recycling to coordinate cellmigration**”** by Zaoui et al., for publication in ***Traffic***. I would like to thank the 2 reviewers for their thoughtful reviews and insightful comments on our work. Briefly, here is an overview of our efforts to address their main comments:

We concluded that Reviewer #1 found our *study convincing and interesting*. However, the reviewer highlighted some of technical issues that needed to be addressed, particularly concerning the live cell imaging.

- We performed additional experiments with faster imaging of single planes to assess

the full range of effects on Rab-4 movements in control and CLIP170KD cell lines. The results are similar to what we have observed in the 3D imaging and are presented in Figure S2A-D.

- We also added immunofluorescence experiments with endogenous CLIP170.
- The other comments were mostly of technical nature and we provide new experiments or explanations in our rebuttal letter to address them.

The Reviewer #2 found that the results of *most of the experiments look straight forward and reasonable* but have one objection with the overall conclusion of the study, as we imply in our conclusions and interpretations that Met recycling to the "leading edge".

- We have decided to replace the term "leading edge" by "cell cortex", which include plasma membrane, cell protrusions, as well as (but not exclusively), the leading edge.
- As requested, we have conducted additional microscopy experiments to further characterized the role of CLIP170 on cell protrusions using Phalloidin staining.
- We also performed immunofluorescence of Arp3 and VASP in control and CLIP-170 KD cells (Fig. S4 A-B).
- I would like to mention that during the revision process, we had also conducted experiments with another CLIP-170 siRNA (smart pool from Dharmacon). We show the main results in Figure R2 for the reviewer.
- We also demonstrate the specificity of our Met antibody with cell lines infected with lenti-*CRISPR* sgRNA targeting Met (Figure R3).

In summary, we have attempted to address all the reviewers' comments, with many new experimental data now added to the paper, as you will see in my rebuttal letter. I am confident the manuscript is significantly improved and will now be in an acceptable format for publication in ***Traffic***.

Thank you for your kind

consideration. Yours sincerely,

Morag Park, Ph.D., *F.R.S.C.*
Director, Goodman Cancer
Research Centre Diane and Sal
Guerrera Chair in Cancer
Genetics James McGill
Professor
Tel. 514 398 5749
Fax. 514 398 6769
Email: morag.park@mcgill.ca

Referees Comments to the Authors

Referee 1:

Comments to the Author

In this work, the authors identify a new interaction between CLIP170, GGA3 and the Met receptor following stimulation of cells with HGF, and demonstrate that this is crucial for recycling the receptor, and for cell migration. This is convincingly shown using a range of complementary techniques. Importantly, they show that other depletion of other microtubule +TIPs does not affect this trafficking, and that defects in recycling after CLIP170 depletion are not due to alterations in MT dynamics (although those do occur). While much of the data are convincing, there are a number of technical issues that need to

be addressed, particularly concerning the live cell imaging.

We thank this reviewer for the critical assessment of our work and for suggesting additional experiments to improve our study.

1. The authors have spent a great deal of effort analysing the effects of various manipulations on GFP-Rab4 vesicle movement, since they have previously shown that a significant proportion of the Met receptor resides in these organelles 20 mins after HGF uptake. My understanding of the methods used is that a complete z-stack is taken every 4 seconds, meaning that there is a time interval of 4 sec between equivalent slices. Furthermore, it is also not clear whether the tracking software will be able to track all GFP-Rab4 structures, regardless of size. This raises the real possibility that only the slower moving, larger Rab4 vesicles are tracked by the software, since faster ones move too far between frames and smaller ones could be missed altogether. In that case, the analysis is performed on only a subset of motile events. Although this doesn't detract from the observations that the Rab4 motility they measure is greatly affected by CLIP170 depletion (etc), it is important to know how complete this data set is. Their previous work used much faster imaging of single planes, rather than slow imaging in 3D, and could be used to assess the full range of effects on movements in one condition: plus/minus CLIP170.

We had already performed faster imaging of single planes to assess the full range of effects on Rab-4 movements in control and CLIP170KD cell lines. The results are similar to what is observed in the 3D imaging (Fig. 2A-E). The loss of CLIP-170 resulted in the failure of most Rab4-positive vesicles to reach the cell periphery and they are instead, localized to perinuclear compartments. These consistent results have been added in Figure S2A-D. The percentage of directed movement and speed of vesicles were analyzed using one plan imaging (A-B) and spinning disc imaging (C-D).

2. More detail should be provided on the imaging system used for live cell imaging, in particular the illumination source and level of photodamage. Another concern is how well controlled the level of GFP-Rab4 expression is between movies, as they vary considerably as presented. Rab overexpression can give major artefacts, including enlarged endosomes and altered motility, so it is important to know how over-expressed it is here. In addition, it is very hard to make out the Rab4 labelling, as each image is shown in green/black rather than greyscale, and always have the tracking data superimposed. The authors should also show controls to demonstrate that microtubule organisation and cell morphology is not affected by their manipulations, as many of the depleted cells have a "fried egg" appearance in the Rab4 images.

We have added more details in the Materials and Methods section. Briefly, for the movement of vesicles, the images in 3D (X, Y, Z) of GFP-Rab4 expressing cells were acquired at 4 seconds intervals for 2 minutes, 10 sections, and 0.5 µm spacing; parameters varied slightly in some experiments. Exposer time was fixed between 500 ms to 900 ms to avoid any over saturation and reduce the phototoxicity.

We also provide to the reviewer representative immunofluorescence images of the microtubule (MT) cytoskeleton of GFP and GRP-Rab4 expressing cells, showing that the MT network is not significantly altered by Rab4 ectopic expression (Figure R1).

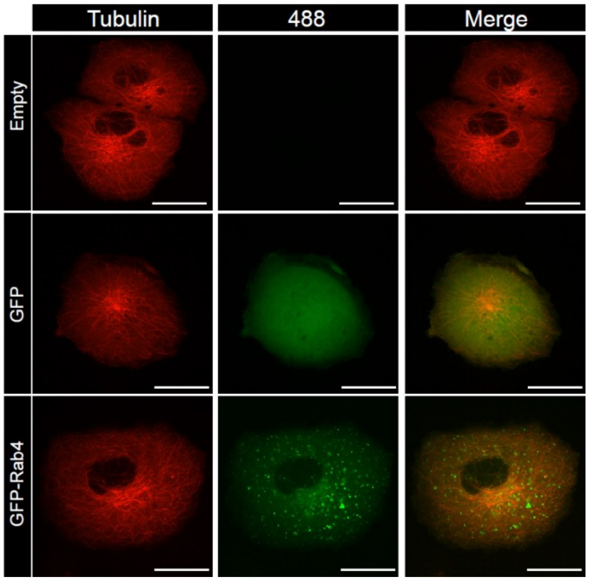

Figure R1. MT network is not altered by Rab4 ectopic expression. Representative images of SKBr3 cells transfected with GFP or GFP-Rab4. Scale bar = 10µm.

3. On p3 is it stated that "Treatment of cells with nocodazole or taxol abrogated HGF-dependent Met recycling from endosomes back to the leading edge". However, from the methods, it is not clear at all how this IF-based recycling assay is quantitated. How is the intensity of the plasma membrane staining distinguished from the endosomal staining, and how is it determined? How is the ratio generated? Or is it simply the ratio of peripheral staining vs. central staining? If so, say that, and explain how the two regions are differentiated, and if the area sampled (i.e. number of pixels) is taken into account. By the way, the text used to describe the analysis is identical to their previous paper (Parachoniak et al., 2011).

The localization of Met to the plasma membrane was determined with the tubulin staining, which delimits the cell cortex. These observations were further substantiated in Fig1D, with subcellular fractionations of PM/lamellipodia and cell body-enriched fractions, showing that Met protein levels are decreased in the PM/lamellipodia fraction of CLIP-170-depleted cells. The figure R2 (A-C) clearly shows that Met is enriched at the cell cortex in control cells while Met is localized in the cytoplasm in CLIP170KD cells.

We have added specification in the Materials and Methods section, as requested by the reviewer. Briefly, MetaMorph® software was used for object-based colocalization measurements. Images were smoothed with a 3 x 3 low-pass filter, and endosomes were identified and counted using size estimates and intensity thresholds in each image set. Binary images were created for each set of endosomal spots and were combined pair- wise to give only the ‘‘colocalized’’ spots. The minimum spot size was set to remove any small spots due to partial, and likely random, overlap of spots. Results were logged into Excel for analysis. Values for all analyses including colocalization and vesicle counting represent mean value ± SEM.

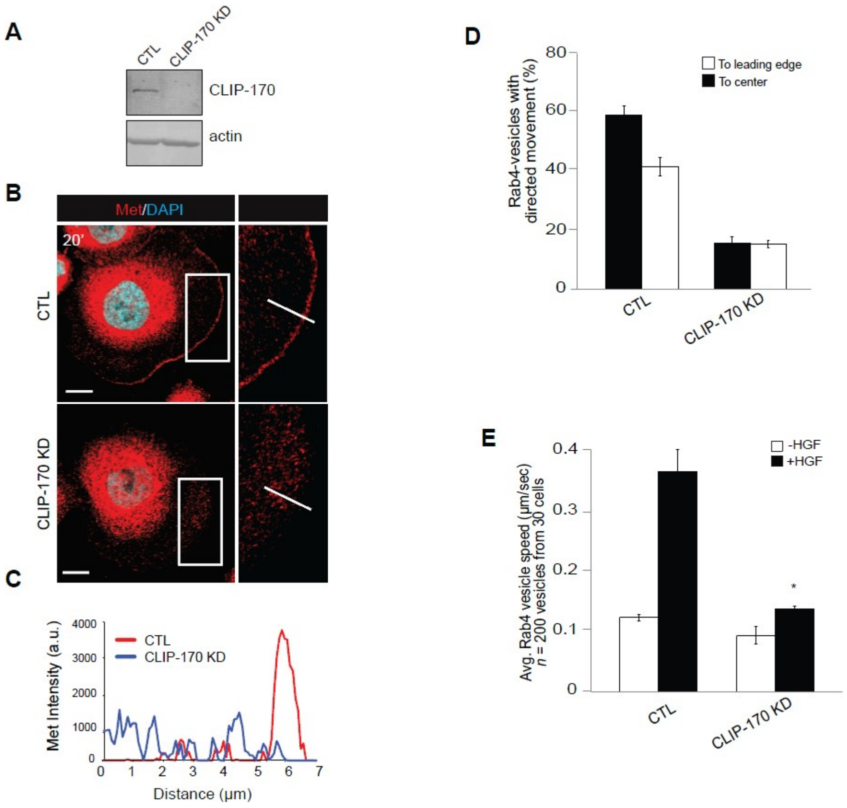

Figure R2. CLIP-170 is required for HGF-induced movement of Rab4-positive vesicles and Met recycling. A) SKBr3 cells transfected with CTL or CLIP-170 (smart pool) siRNA were lysed and proteins were separated by SDS-PAGE and immunoblotted as shown. B) Representative images of SKBr3 cells transfected with CTL or CLIP-170 siRNAs. Insets show enlargement from the leading edge. C) Graph show the distribution of Met staining from the nucleus to the cell cortex. D-E) SKBr3 cells co-transfected with GFP-Rab4 and CTL or CLIP-170 siRNAs, were treated with 0.5 nM HGF (20 minutes). Individual Rab4-positive vesicles were tracked over time. The percentage of directed movement (D) and speed of vesicles (E) were analysed. Scale bar = 10µm. * $p < 0.05$.

The IF recycling assay is backed up by a cell fractionation approach (Fig. 1D). However, a single blot is shown, without any quantitation. Can the authors quantitate this change in 3 independent experiments?

We performed quantification of 3 independent experiments and added a graph below panel (Fig. 1D).

4. In the same experiment, Fig. S1A shows that the level of Met drops dramatically in taxol-treated cells. What is going on?

We thank the reviewer to pointing that out. This image was not the most representative. We have changed the picture in Fig. S1A and similarly to what is shown in Fig. 1A, the level of Met level is not altered following taxol treatment. These observations are also in agreement with work of another group (1).

5. In Fig. S1B, it would be much better to show the effect of EB1 KD on native CLIP170 distribution, not on exogenously-expressed RFP-CLIP170. The KD image shown has a large CLIP170 aggregate in the centre, but this could be an over-expression artefact.

This is an important point that we had already began to address during the revision period. We now reported that similarly to what we observed with ectopic-CLIP170, EB1 depletion

leads to CLIP-170 delocalization from MTs (Fig. S1C). We have now replaced panel of Fig. S1C with immunofluorescence images showing endogenous CLIP170 localization in control and EB1 KD cells.

6. The authors state on page 4 that they are expressing a dominant negative EB3-GFP, but then contradict this in the text and Fig 2 legend to imply that it inhibits CLIP170 binding simply because it is over-expressed. Please clarify which is correct.

We thank this reviewer for pointing this out and we like to confirm that we used the EB3-GFP construct, that occludes and displaces the plus-end binding of CLIP-170 by inhibiting CAP-GLY-EEY/F interactions as described in (2). We have now clarified the figures legends, results and Materials and Methods sections accordingly.

On p4 and in Fig 2G "the percentage of cells with aggregated…Met-positive vesicles at the MTs plus ends" is assessed. What does this mean? Are they only scored in the vicinity of +ends (and if so, how are the +ends identified), or is this just inaccurate writing? There is no information in the methods on what has been done. To back up this scoring, representative images of each category should be included as supplemental data.

The percentage of cells with aggregated (A), partially aggregated (PA), or dispersed (D) Met, have been determined as previously described (3). The Met-positive vesicles "at the MTs plus ends" have been scored at the vicinity of +end, stained with EB3-GFP. We now provide additional details on the quantification in the Materials and Methods section. In addition, we added representative images for "Aggregated", "Partially aggregated" and "Dispersed" (Fig. S3D).

7. In Fig. 3D it appears that the CLIP170-deltaH interacts better with Met than full length CLIP170. Is that correct? Does that hint at regulation of the interaction via changes in CLIP170 conformation?

This is an excellent comment. Previous work reported that CLIP-170 can fold back upon itself through an interaction between its NH2 and COOH termini. Moreover, it was proposed that CLIP-170 alternates between an active, extended conformation, in which it can interact with MTs, and an inactive, folded conformation, in which it is not bound to other proteins (4). We believe that the deletion of CLIP-170 head domain induces a conformational change where CLIP-170 is probably in an "open" state because this mutant bind strongly to Met. In addition, it possible that removal of CLIP-170 head domain disrupt CLIP-170 interactions with other partners at its NH2 terminus and then, facilitating its interaction with Met. These observations could explain why Met has a better interaction with CLIP-170□H rather than the full length.

8. In the discussion on p 10 the authors state that "The enhanced localization of Met-positive vesicles towards the MT plus-ends following HGF stimulation, increases the stability of MT growth to lamellipodia…". They have not shown this at all, and the two statements are not necessarily linked. In the final paragraph of the discussion they suggest that Met-vesicles bound to CLIP170 are transported by plus-end motors. Do the authors mean that they are first transported with the kinesin, then bind to the +end via CLIP170? The alternative wouldn't work, since if they are already at +TIPs via CLIP170 binding, then there is no MT for a kinesin to move along. It would seem more likely that the +end movement

The reviewer raised a good point, we didn't investigate the kinesins properly. We also replace the statement "stability of MT growth to lamellipodia…" by "modulates cell protrusion dynamics". Thank you for pointing this out, we have corrected these inappropriate terminologies.

Minor points

- The title of Fig. S5 is misleading, as the involvement of Arf6 GTPase has not been directly tested.

We have previously showed that the GGA3 GAT-N194A mutant completely lost its ability to interacts with Arf6. Moreover, we showed that GGA3 GAT-N194A mutant phenocopy the depletion of Arf6 (5, 6). That is why we mention that Arf6 GTPase is not required for the interaction of CLIP-170 and GGA3. However, as the involvement of Arf6 GTPase has not been properly demonstrated in this present work, we changed the title accordingly to" CLIP-170 bound to the GAT domain of GGA3."

- On p 5, it would be better to write "…levels of Met returning to the surface to 15% compared to 42%..." (rather than "by 15%") and also later in the same sentence "…to 7%" rather than "by 7%".

- On p6, Fig 3G should read Fig 3F, and 3H should be 3G.

Thank you for highlighting these points out, we have corrected accordingly.

- In Fig. 4, it is stated that "…the data represent the trajectories of ten cells". Presumably this is 10 cells in 3 repeats, meaning 30 cells (otherwise the numbers in the graphs don't make sense).

We have analyzed 30 cells for each repeat (n=3), for a total of 90 cells. The right panel of Fig. 4C shows the trajectories of 10 cells, as described in figure legend. We have now replaced this panel to show all the analyzed cells in a representative experiment (30 cells).

- The legend title for Fig. 5 is missing a verb. Do they authors mean "…Rab4-positive vesicle movement"?

We have corrected the title accordingly.

- The authors should refer to the reported interaction between cytoplasmic dynein and Rab4, in Bielli et al. (2001). Biochemical and Biophysical Research Communications, 281(5), 1141–1153.

We have added some additional key references from the endocytosis field in the manuscript. We thank the reviewer for suggesting this reference.

Referee: 2

Comments to the Author

MS ID: TRA-18-0759: "CLIP-170 spatially modulates receptor tyrosine kinase recycling to coordinate cell migration."

Kossay Zaoui, Stephanie Duhamel, Christine Anna Parachoniak, and Morag Park

In this manuscript, the authors describe that recycling of the receptor ryrosine kinase hepatocyte growth factor receptor (also known as c-Met) occurs in a microtubule- dependent fashion, and in association with a prominent +tip targeting protein of microtubules known as CLIP-170. Initially after HGF treatment of cells, Met is internalized to end up in Rab4-positive endosomes, which together with GGA3 (Golgi- localized γ-ear-containing Arf-binding protein 3), are recycled back to the plasma membrane. And all this then, the authors propose, promotes HGF-induced signaling to lamellipodia formation and HGF-triggered cell migration.

The manuscript contains quite a lot of data, and the results of most of the experiments look pretty straight forward and reasonable, but I have one major objection with the overall conclusion of the study, which is as follows: The authors imply in their conclusions and in the interpretation of their results in general that it is important for cells to position their

receptors (in this case c-Met receptor) at what they call the "leading edge", so at the plasma membrane tips of in this case lamellipodial protrusion and/or membrane ruffles. This view can be readily deduced from the summary model on page 34 of the submitted manuscript (step 5), in which Met-receptor dimers localize at the tips of membrane protrusions, quasi ahead of the lamellipodial actin network and nascent adhesions. In fact, this is by no means realistic, and indeed the data don't show this either, and in addition, I have never seen clear-cut data from any other lab that has shown such a localization for RTKs, such as Met, EGFR or PDGFR. Instead, it was previously shown by TIRF microscopy, for instance, that EGFR can accumulate at clathrin-coated structures, and its endocytosis coincide with clathrin-mediated endocytosis (see e.g. Benesch et al., J Cell Sci 118 (Pt14):3103-15), so can occur scattered over the entire cell surface.

So I would agree with the authors that Met trafficking on Rab4-positive vesicles may occur to the plasma membrane, but I would refrain from stating that this occurs in a directional fashion towards the leading edge, and that this then is relevant for accumulation of the receptor at the leading edge membrane of lamellipodial protrusions, as no evidence for this is provided!

These are excellent comments. We understand the reviewer reserve with the term ''leading edge'' and have decided to replace ''leading edge'' by ''cell cortex", which include plasma membrane, cell protrusions, as well as (but not exclusively), the leading edge. As per the suggestions of this reviewer, we also conducted additional experiments that allow us to broaden our conclusions.

Specific Critique:

1) In Figure 1, we are looking at the distribution of microtubules and Met by indirect IF, but no evidence is provided that the spots we are looking (in red) actually correspond to Met-containing structures. Can the authors provide a control experiment to prove that the antibody staining is specific for Met, such as showing that in Met knockdown or -KO cells, no such staining is observed?

We have generated HeLa cells with stable knock out (KO) of Met using the lentiCRISPR v2 system. Immunofluorescence and western blot of control and Met-KO cell clearly show the specificity our Met antibody; anti-Met (AF276) from R&D Systems.

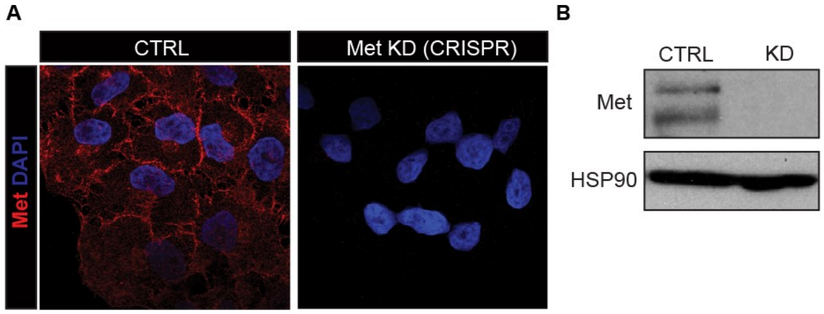

Figure R3. Specificity of Met antibody. A) Representative images of HeLa cells infected with lenti-CRISPR sgRNA targeting Met (KD) or control empty-vector (CTRL), stained with Met antibody (anti-Met (AF276 from R&D Systems). B) HeLa cells infected with lenti-CRISPR sgRNA targeting Met or control empty-vector were lysed and separated by SDS-PAGE and immunoblotted as shown.

2) In all Figures, the authors jump forth and back between cell types, so HeLa-cells in Figure 1, SKBr3 in Figure 2, HeLa again in Figure 3 and so on. I don't know which cell type was used in 4, Figure 5 is SKBr3 again, HEK293 in Figure 6, and so on and so forth also throughout the Supplement. No explanation is provided why the authors have chosen a specific cell type for a given experiment, so this looks a bit like a wild mixture of data that has been assembled together. I would recommend the authors to clearly state in the manuscript why a given cell type was chosen and is shown in a given Figure to make this

clear.

We have now clearly indicated that the migration assay in Fig.4 have been performed with HeLa cells, our apologies it was an oversight. We have performed our experiments in more than one cells type to further exemplified that our results are observed in more than one cell type. HeLa cells are the most used and characterized for recycling experiments by our group and others as well (1, 5-9). We studied the role of CLIP-170 in Met trafficking in HeLa cells (Fig. 1, 3, S1-S3) and validate the strict requirement of CLIP-170 for HGF-induced movement of Rab4-positive vesicles and Met recycling in the SKBR3 cells (Fig. 2,5, S1-S3). Similarly, HEK293T and HeLa cells have been used for the biochemistry experiments. In figure 6A, co-immunoprecipitation of HA-CLIP-170 and Myc-GGA -1, -2 or, -3 proteins were performed in HEK293T. The interaction of CLIP-170 with GGA-3 was then reconfirmed in HeLa cells (Fig. 6B).

3) As far as I understand, much of the conclusions are currently building on RNAi-mediated knockdown of CLIP170 and other factors, but it is not clear if the authors have convincingly shown by Western that the rescue construct (of CLIP170) is re-expressed; I could only find reduction of CLIP170 expression in the knockdown. I guess Figure S2A shows the reduction of CLIP170 expression in the knockdown situation, but the rescue could not be found.

We have now added clarification in the figure legend of Fig.S2E (Fig.S2A in the previous version of the manuscript). The cells used for this experiment are the same one as Fig S1D, where the efficiency of the rescue of CLIP170 in the SKBR3-CLIP170KD cells is clearly shown by WB.

Moreover, it is a bit dangerous to solely rely on just one type of siRNA, as normally, at least two independent siRNAs are used for documenting the consequences of knockdown of a given factor. I would recommend repeating experiments with a second, independent siRNA sequence, and/or confirm the functional data with an alternative approach of interference with gene function, such as CRISPR/Cas9.

We agree with the reviewer that at least two independent siRNAs should be used. We had already preformed experiments with another siRNA (smart pool from Dharmacon). We show the main results in Figure R2 for the reviewer.

4) The protrusion analysis shown in Supplementary Figure S4 does not make much sense to me. The kymography is OK, in principle, but the contrast in the phase-contrast images is very poor. Most importantly, though, the "width" and "length" analysis (see Figure S4B) does not have anything to do with a reasonable classification of lamellipodial protrusions, as the authors are simply measuring the distance between periphery and nucleus (and for the width a distance orthogonal to that), without any distinction between different subcellular areas, such as lamellipodium versus lamella further proximal to the lamellipodium etc. The type of analysis provided here can perhaps give some indirect information on how large the area is the cells are adopting (i.-e. cell spreading efficiency, although there is much more elegant ways to do this), but it can by no means provide any information on protrusion parameters. For the latter, the authors would have to perform actin filament stainings, e.g. using fluorescent phalloidin, and then analyze the dimension of lamellipodia and/or other types of protrusions at the cell periphery. With the poor quality phase contrast images shown here, no clear classification of protrusions is possible, and hence very limited information can be obtained from such analyses.

We thank the reviewer for these pertinent comments. To further insights into the role of CLIP170 on cell protrusion, we have conducted additional microscopy experiments. We added immunofluorescence of Arp3 and VASP in control and CLIP-170 KD cells (Fig. S4 A-B). VASP localizes to waving protrusion of the leading edge (10). As requested by the reviewer, we also preformed actin filament staining, with fluorescent phalloidin and analyzed the dimension of the cell protrusions at the cell periphery, as well as lamellipodia and filopodia (Figures S4C-E).

5) In Figure 5, the authors also describe effects of CLIP170 knockdown on microtubule dynamics, such as growth or shortening rate and rescue or catastrophy

frequencies. First, the table shown in Figure 5A indicates that the differences with many of these parameters are quite small.

More importantly though, the authors also claim that the differences observed were dependent on HGF, which would be quite astonishing to me…(see middle of 2nd para on the left)!

However, I can't discern where evidence for this is shown, as all conditions in Table 1 are either "with HGF" or "with EGF", but no data for the absence of those growth factors are shown, correct?

We are now showing our results in graph instead of a table. The p-value are clearly presented, and we have added the condition without growth factor.

Minor comments:

6) In Figure 2J, I guess the authors meant to say that after 13 minutes of HGF treatment, levels of recycled Met were reduced to 7% (not "by 7%") as compared to 55% in the control!

7) In Figure 3D, Lysate is mis-labelled as "Lystae 10%!

These are excellent points and we have corrected the accordingly.

8) At the end of the discussion, the authors propose that Met-positive vesicles that are bound to CLIP-170 are transported by plus-end-directed kinesins, although in my view, CLIP170-mediated tracking on microtubule tips alone might be sufficient for the observations made in this manuscript. I feel that without any evidence for the involvement of kinesins, such statements should clearly be avoided!

Thank you for pointing this out, we have changed the discussion consequently.

REFERENCE

1. Menard L, Parker PJ, Kermorgant S. Receptor tyrosine kinase c-Met controls the cytoskeletonfromdifferentendosomesviadifferentpathways.Nat Commun2014;5:3907.

2. Komarova Y, Lansbergen G, Galjart N, Grosveld F, Borisy GG, Akhmanova A. EB1 and EB3 control CLIP dissociation from the ends of growing microtubules. Mol Biol Cell 2005;16(11):5334-5345.

3. Lomakin AJ, Semenova I, Zaliapin I, Kraikivski P, Nadezhdina E, Slepchenko BM, Akhmanova A, Rodionov V. CLIP-170-dependent capture of membrane organelles by microtubules initiates minus-end directed transport. Dev Cell2009;17(3):323-333.

4. Lansbergen G, Komarova Y, Modesti M, Wyman C, Hoogenraad CC, Goodson HV, Lemaitre RP, Drechsel DN, van Munster E, Gadella TW, Jr., Grosveld F, Galjart N, Borisy GG, Akhmanova A. Conformational changes in CLIP-170 regulate its binding to microtubules and dynactin localization. J Cell Biol 2004;166(7):1003-1014.

5. Parachoniak CA, Luo Y, Abella JV, Keen JH, Park M. GGA3 functions as a switch to promote Met receptor recycling, essential for sustained ERK and cell

migration. Dev Cell 2011;20(6):751-763.

6. Puertollano R, Randazzo PA, Presley JF, Hartnell LM, Bonifacino JS. The GGAs promote ARF-dependent recruitment of clathrin to the TGN. Cell 2001;105(1):93-102.

7. Abella JV, Parachoniak CA, Sangwan V, Park M. Dorsal ruffle microdomains potentiate Met receptor tyrosine kinase signaling and down-regulation. J Biol Chem 2010;285(32):24956- 24967.

8. Ratcliffe CD, Sahgal P, Parachoniak CA, Ivaska J, Park M. Regulation of Cell Migration and beta1 Integrin Trafficking by the Endosomal Adaptor GGA3. Traffic 2016;17(6):670-688.

9. Abella JV, Vaillancourt R, Frigault MM, Ponzo MG, Zuo D, Sangwan V, Larose L, Park M. The Gab1 scaffold regulates RTK-dependent dorsal ruffle formation through the adaptor Nck. J Cell Sci 2010;123(Pt8):1306-1319.

10. Barnhart EL, Allard J, Lou SS, Theriot JA, Mogilner A. Adhesion-Dependent Wave Generation in Crawling Cells. Curr Biol 2017;27(1):27-38.

Decision and Reviews

Dear Dr. Park,

Thank you for submitting your revised manuscript “CLIP-170 spatially modulates receptor tyrosine kinase recycling to coordinate cell migration” to Traffic. I asked referee 1 to review the revised paper. He/she did not have comments for the referee, but made the recommendation in confidential comments that the paper be accepted at this time. I agree with the referee and am pleased to accept this paper.

Sincerely,

Trina A. Schroer, PhD
Co-Editor

___

Referee's Comments to the Authors

Referee: 2

Comments to the Author
(There are no comments.)

Author Rebuttal

Dear Dr. Schroer,

I am pleased to submit the final version of our manuscript (TRA-18-0759.R2), entitled “CLIP-170 spatially modulates receptor tyrosine kinase recycling to coordinate cell migration” by Zaoui et al., for publication in Traffic.

The referee did not have comments and recommended that the paper be accepted at this time.

As requested, we have reformatted the references to the JAMA reference style and list them in the order cited in the reference section.

Thank you for your kind consideration.

Sincerely,

Morag Park
